# De novo transcriptome assembly for the five major organs of *Zanthoxylum armatum* and the identification of genes involved in terpenoid compound and fatty acid metabolism

**DOI:** 10.1186/s12864-020-6521-4

**Published:** 2020-01-28

**Authors:** Wen-Kai Hui, Fei-Yan Zhao, Jing-Yan Wang, Xiao-Yang Chen, Jue-Wei Li, Yu Zhong, Hong-Yun Li, Jun-Xing Zheng, Liang-Zhen Zhang, Qing-Min Que, Ai-Min Wu, Wei Gong

**Affiliations:** 1 Key Laboratory of Ecological Forestry Engineering of Sichuan Province, College of Forestry, Sichuan Agricultural University, Chengdu, 611130 China; 2 Guangdong Key Laboratory for Innovative Development and Utilization of Forest Plant Germplasm, State Key Laboratory for Conservation and Utilization of Subtropical Agro-Bioresources, College of Forestry and Landscape Architecture, South China Agricultural University, Guangzhou, 510642 China; 3 Agricultural Technology Extension Center in Yantan District, Zigong, 643030 China

**Keywords:** *Zanthoxylum armatum*, De novo transcriptome, Aromatic compounds, Fatty acid

## Abstract

**Background:**

*Zanthoxylum armatum* (*Z*. *armatum*) is a highly economically important tree that presents a special numbing taste. However, the underlying regulatory mechanism of the numbing taste remains poorly understood. Thus, the elucidation of the key genes associated with numbing taste biosynthesis pathways is critical for providing genetic information on *Z*. *armatum*and the breeding of high-quality germplasms of this species.

**Results:**

Here, de novo transcriptome assembly was performed for the five major organs of *Z. armatum*, including the roots, stems, leaf buds, mature leaves and fruits. A total of 111,318 unigenes were generated with an average length of 1014 bp. Additionally, a large number of SSRs were obtained to improve our understanding of the phylogeny and genetics of *Z. armatum*. The organ-specific unigenes of the five major samples were screened and annotated via GO and KEGG enrichment analysis. A total of 53 and 34 unigenes that were exclusively upregulated in fruit samples were identified as candidate unigenes for terpenoid biosynthesis or fatty acid biosynthesis, elongation and degradation pathways, respectively. Moreover, 40 days after fertilization (Fr4 stage) could be an important period for the accumulation of terpenoid compounds during the fruit development and maturation of *Z. armatum*. The Fr4 stage could be a key point at which the first few steps of the fatty acid biosynthesis process are promoted, and the catalysis of subsequent reactions could be significantly induced at 62 days after fertilization (Fr6 stage).

**Conclusions:**

The present study realized de novo transcriptome assembly for the five major organs of *Z. armatum*. To the best of our knowledge, this study provides the first comprehensive analysis revealing the genes underlying the special numbing taste of *Z. armatum.* The assembled transcriptome profiles expand the available genetic information on this species and will contribute to gene functional studies, which will aid in the engineering of high-quality cultivars of *Z. armatum*.

## Background

*Zanthoxylum armatum* (Rutaceae), commonly known as green Sichuan pepper, is one of the most economically important trees in Sichuan Province and is widely distributed in most parts of southwest China and some parts of southeast Asia [1]. The production of *Z*. *armatum* is currently a billions of dollars commercially, and this species has a long history of cultivation in China because it is one of the eight main spices used in Chinese cuisine and is an essential ingredient in Sichuan hot-pot, with a special numbing taste [2]. However, the underlying regulatory mechanism of the genes associated with the numbing taste remains poorly understood. *Z*. *armatum* is also an important medicinal plant prioritized by the governments of some countries for economic development [3–5]. Its fruit berries can be used for the treatment of abdominal pain, rheumatism, and skin diseases, and as a carminative and antispasmodic [3]. The seeds are employed as an aromatic tonic for conditions such as fever, dyspepsia, toothache, and stomach ache [3, 4]. Thus, the elucidation of the keygenes associated with numbing taste biosynthesis pathways, especially those responsible for the accumulation of the main compounds involved, is critical for revealing genetic information for this species and breeding high-quality *Z*. *armatum* germplasms.

In previous studies, the numbing taste was found to be associated with the presence of volatile oils, alkaloids, coumarins, acid amide phenol components and so on [6, 7], which accumulate at high levels in the fruits, leaves, stems, and roots of *Z*. *armatum*, especially in the pericarps [8]. More than 140 components related to aromatic compounds and fatty acid biosynthesis have been gradually identified in different tissues of *Z*. *armatum* [9]. Various terpenoid substances are among the main components associated with the numbing taste of *Z*. *armatum*, including linalool (29.30%), limonene (14.30%), myrcene (6.02%), cineole (1.32%) and so on [7, 10]. Terpenoids are a large category of necessary secondary metabolites in plants, includingmonoterpenes, diterpenes, sesquiterpenes, triterpenoids and other terpenoid-quinone compounds [11]. All of these downstream products accumulate from the terpenoid backbone biosynthesis pathway. The five key enzymes, acetoacetyl-CoA thiolase 2 (ACAT2), hydroxy-methyl-glutaryl-CoA synthase (HMGS), hydroxy-methyl-glutaryl-CoA reductase (HMGR), mevalonate kinase (MVK), and diphosphomevalonate decarboxylase (MVD) are required for the only pathway producing isopentenyl-*PP* upstream of terpenoid backbone biosynthesis [12]. Subsequently, the production of dimethylallyl-*PP* from isopentenyl-*PP* can be catalysed by isopentenyl-diphosphate delta-isomerase (IDI) [13]. Additionally, dimethylallyl-*PP* can be produced via the methylerythritol phosphate (MEP) pathway. Next, isopentenyl-*PP* and dimethylallyl-*PP* are transformed into farnesyl-*PP* and geranyl-geranyl-*PP*, which are then used to produce various terpenoids, catalysed by different diphosphate synthases [14]. Fatty acids are also important productsfound in the fruit of *Z*. *armatum*. Abundant unsaturated fatty acids have been detected in the seeds of *Zanthoxylum* (approximately 25%), among which linolenic acid, linoleic acid and oleic acid account for 78.63% (mass percentage) of the total, while the main saturated fatty acid is hexadecanoic acid (13.18%) [15, 16]. The production of fatty acids is involved in the de novo fatty acid biosynthesis, elongation and degradation pathways [16]. In the de novo fatty acid biosynthesis pathways, acetyl-CoA is produced and used to form malonyl-ACP, catalysed by acetyl-CoA carboxylase (ACC) and malonyl-transferase (MCMT). Subsequently, malonyl-ACP can be converted to a long-chain acyl-ACP catalysed by several enzymes with an acyl carrier protein (ACP) as a co-factor [17]. Three main enzymes that play an important role during this series of reactions include ketoacyl-ACP synthase II (KASII), ketoacyl-ACP synthase I (KASI), and oxoacyl-acyl-carrier protein reductase (FabG) [18]. Moreover, ACP desaturase 5 (AAD5), also known asstearoyl-ACP desaturase (SAD), removes two hydrogen atoms from stearic acid to form oleic acid in unsaturated fatty acid production [19]. Finally, *N*-acyl-ACP (C8 to C18) is hydrolysed to release free fatty acids [20]. Thus far, many studies on *Zanthoxylum* have mainly dealt with plant breeding, physiological investigations, tissue culture and chemical component identification and extraction in the fruits [8, 21]. A recent study identified the key genes in the synthesis pathway of volatile terpenoids in the fruit of *Zanthoxylum bungeanum* (red Sichuan pepper) [22]. However, the crucial genes involved in the biosynthesis of terpenoid compounds and fatty acids have not been reported in *Z. armatum*.

Comparative transcriptome analysis is an important method for rapidly obtaining a large amount of genetic information and putative candidate genes related to target traits by examining different tissue samples. Although transcriptome sequencing analyses have rarely been reported in *Z. armatum*, they have been conducted frequently in other tree species. The de novo transcriptome sequencing of eight major organs of *Plukenetia volubilis* was performed to identify candidate genes involved in α-linolenic acid metabolism, thereby expanding the genetic information available for functional genome studies of *P. volubilis* [20]. Through the transcriptome sequencing analysis of roots, stems, leaves, arils and kernel samples of two *Torreya grandis* cultivars, six candidate unigenes encoding sciadonic acid elongase and desaturases were identified to improve the understanding of the molecular mechanisms responsible for de novo fatty acid biosynthesis in gymnosperm species [16]. Additionally, Wang et al. [21] conducted a detailed transcriptional sequencing analysis of two orange varieties at different fruit development stages to elucidate the underlying regulatory mechanism of sucrose and citrate accumulation in the ripening of the fruits, especially during the fruit delayed-harvest stage. Zhang et al. [23] explored the key regulatory factors involved in starch and sucrose metabolism in *Castanea mollissima* via transcriptome sequencing of seeds at various developmental stages. However, only a single study has reported transcriptome profiles obtained using a mixture of the leaves and inflorescences of *Z. armatum* to isolate the viruses associated with flower yellowing disease in recent years [24].

In the present study, the de novo transcriptome sequencing of five major organs was performed using the Illumina HiSeq 4000 platform. The key candidate genes associated with terpenoid compounds and fatty acid biosynthesis and metabolism were identified from the RNA-seq dataset in *Z. armatum*. Furthermore, samples from different stages in the fruit development and maturation process were selected to identify the expression patterns of the key candidate genes through qRT-PCR analysis. To the best of our knowledge, ours is first comprehensive analysis to reveal the genes underlying the special numbing taste of *Z. armatum*. The assembled transcriptome profiles expand the available genetic information for *Z. armatum* and provide an improved understanding for gene function studies, which will aid in the engineering of high-quality varieties of *Z. armatum*.

## Results

### Transcriptome sequencing and de novo assembly of *Z. armatum*

The total RNA of five major sample types, including roots (Ro), stems (St), leaf buds (LB), mature leaves (ML) and fruits (Fr), was isolated to construct the comprehensive transcriptome of *Z. armatum*. The quality of the RNA was determined using the OD260/OD280 ratio (1.88–2.21) and RIN (7.60–10.00) (Additional file 1: Table S1). A total of 126.89 G of paired-end raw reads were produced by transcriptome sequencing. After trimming adapters and low-quality bases, 7.92–9.91 G of clean bases were produced from 15 cDNA libraries in this study (Additional file 2: Table S2). The error rate of RNA-seq was only approximately 0.02%, all of the Q30 values were greater than 93.90%, and the GC content was above 43% in each sample. In addition, an assembly of 350,625 transcripts was achieved, with a mean length of 1219 bp for the *Z. armatum* transcriptome (Additional file 3: Figure S1a). The longest transcript of each gene was chosen from the assembly results as the candidate unigene. Finally, a set of 111,318 unigenes was obtained in the present study (Additional file 3: Figure S1b). The length of the unigenes ranged from 301 to 17,299 bp, with an average of 1014 bp, and the lengths of more than half of the unigenes in the total assembly were greater than 1454 bp (*N*50 = 1454).

### Identification of simple sequence repeats

**Fig. 1:**
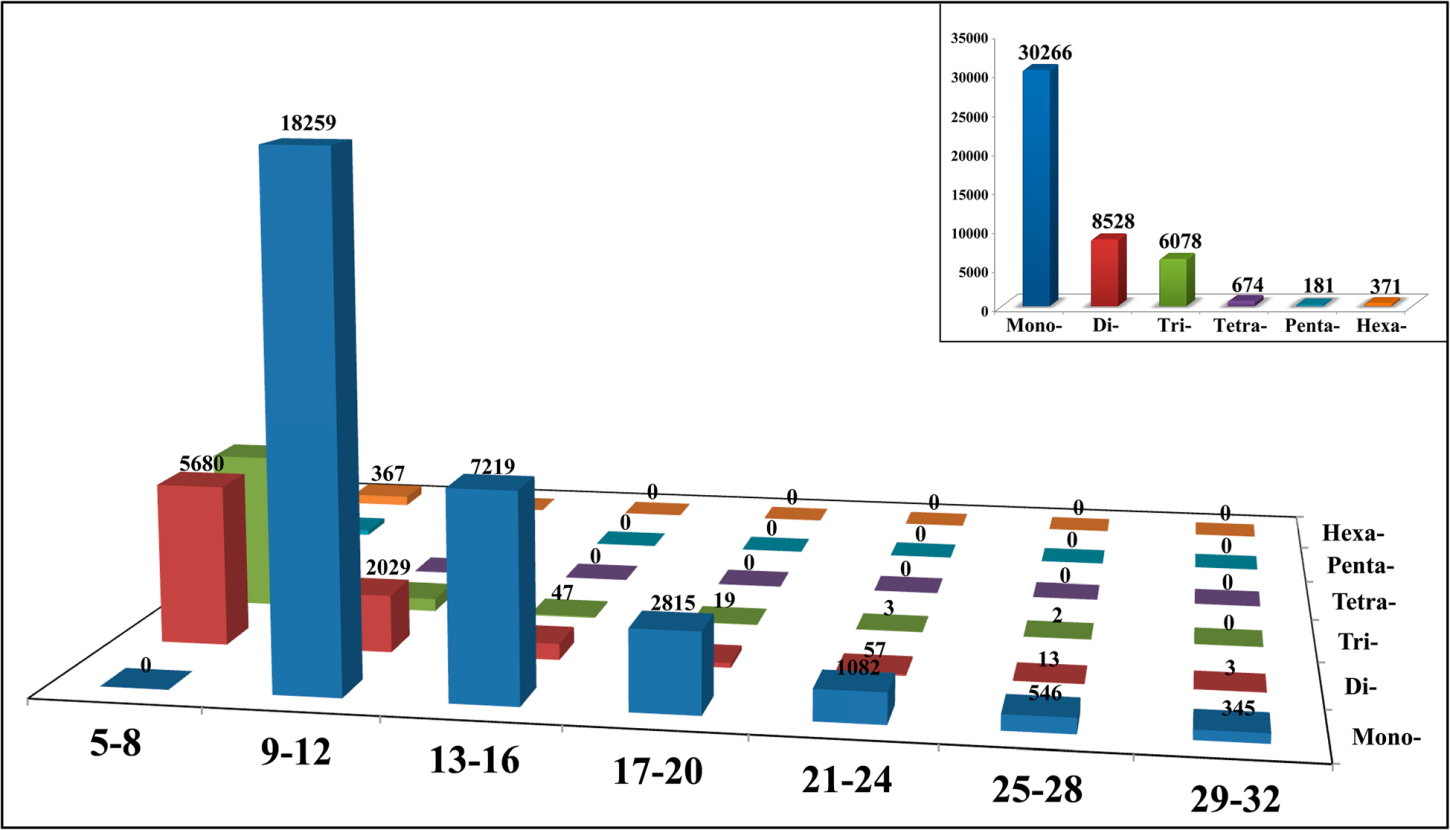
The distribution of SSRs in *Z. armatum*. The insert shown the distribution of the total number of SSRs in mono-, di-, tri-, tetra-, penta- and hexa-nucleotide repeats

In recent years, various molecular markers have been widely developed in different plants to construct plant genetic maps, perform gene localization, determine hybrid purity, and examine other aspects. To obtain abundant molecular markers for the genetic analysis and marker-assisted selection breeding of *Z. armatum*, simple sequence repeats (SSRs) were identified in our transcriptome (Fig. 1). Mono- to hexanucleotide SSRs were identified using MISA software (Fig. 1, Additional file 4: Table S3). A total of 46,098 SSR loci were characterized, among which mononucleotide repeats were the most abundant (30,266, 65.66%), followed by dinucleotides (8528, 18.50%). Moreover, at least 9–12 repeats were detected among the monomer nucleotide SSRs. However, di- to hexanucleotide SSRs were explored mostly in the context of 5 to 8 repeats. These results indicated that high variation might exist in *Z. armatum.*

### Gene functional annotation

After assembly, all the unigenes were subjected to BLASTx analysis against five public databases, including NR, NT, KO, Pfam, and KOG, to characterize their gene functions (Additional file 5: Figure S2, Table 1). A total of 73,426 (65.96%) unigenes were annotated in at least one database. The highest annotation rate was obtained in the NR database, which assigned 61,598 (55.34%) unigenes. A total of 34,930 (31.38%) and 16,316 (14.66%) unigenes were annotated with the Pfam and KOG databases, respectively. The top-scoring BLASTx hits against the NR protein database showed that the E-value distribution presented a comparable pattern with 50.70% of the mapped sequences with high homologies (< 1e-45), whereas the E-values for 49.30% of the homologous sequences ranged between 1e-5 and 1e-45 (Additional file 5: Figure S2a). The distribution of sequence similarity showed that 53.90% of the mapped sequences presented similarities higher than 80%, while 10.50% of the hits exhibited similarities lower than 60% (Additional file 5: Figure S2b). Additionally, the species distribution of the NR BLASTx matches showed that the top three species were *Citrus clementina* (21.80%), *Citrus unshiu* (19.00%) and *Citrus sinensis* (16.10%), and all of these species and *Z. armatum* belonged to Rutaceae (Additional file 5: Figure S2d). All the above results indicated that a high-quality annotation was obtained in the present study.

**Table 1:** Summary of the annotation about the *Zanthoxylum armatum* transcriptome

Items	Number of Unigenes	Percentage (%)
Annotated in NR	61,598	55.34
Annotated in NT	55,465	49.83
Annotated in KO	24,137	21.68
Annotated in Pfam	34,930	31.38
Annotated in KOG	16,316	14.66
Annotated in all Databases	4656	4.18
Annotated in at least one Database	73,426	65.96
Total Unigenes	111,318	100

Moreover, 34,930 (31.37%) unigenes were annotated with 55 Gene Ontology (GO) terms, including 26 terms related to biological processes, 19 terms related to cellular components, and 10 terms associated with molecular functions (Additional file 6: Figure S3). Under the biological process, cellular component and molecular function categories, the predominant groups were assigned to the cellular process (GO: 0009987) and metabolic process (GO: 0008152); cell (GO: 0005623) and cell part (GO: 0044464); and binding (GO: 0005488) and catalytic activity (GO: 0003824) terms, respectively. To further understand the biological functions and interactions of the unigenes, they were also classified into metabolic pathways using the Kyoto Encyclopedia of Genes and Genomes (KEGG). A total of 24,137 unigenes (21.68%) were assigned to 19 categories divided into five clusters, including cellular processes, environmental information processing, genetic information processing, metabolism and organismal systems (Additional file 7: Figure S4). Among the KEGG pathways (Additional file 8: Table S4), the top five categories were translation (2916 unigenes), carbohydrate metabolism (2226 unigenes), overview (1771 unigenes), folding and degradation (1659 unigenes), and amino acid metabolism (1394 unigenes). All of these results showed that the investigated samples were characterized by active cell development and differentiation. Four hundred and twenty two unigenes were annotated with metabolism of terpenoids and polyketides, and 376 of which were related to monoterpenoid, diterpenoid, sesquiterpenoid and triterpenoid, limonene and pinene, carotenoid, and terpenoid backbone biosynthesis pathways. Additionally, 314 unigenes were involved in fatty acid biosynthesis and metabolism. These unigenes were the main substances associated with fatty acid and aromatic compound accumulation. It is worth noting that all of these putative genes were differentially expressed among the five different samples in this study (Additional file 9: Figure S5, Additional file 10: Figure S6), and more unigenes were up-regulation in the fruit samples. This suggested that some organ-specific unigenes might be existed in various tissues, and the fruit could be the mainly organs to accumulate the numbing-taste related compounds.

### Investigation of organ-specific unigenes

The Pearson correlation analysis revealed that all three independent biological replicates of each sample presented good reproducibility in the present study, and the stem samples showed the highest correlation coefficient among all of the investigated organs (Additional file 11: Figure S7). To further analyse the characteristics of the genes related to the different organs, the organ-specific unigenes of the five major samples were screened on the basis of a *p* value < 0.05 and |log2(fold change)| >5. The expression values (FPKM) for each comparison were for one organ and the sum of other organs. The investigation of the organ-specific unigenes expressed in each organ showed that 4970, 90, 2314, 1955, and 650 unigenes were specifically found in the roots, stems, leaf buds, mature leaves, and fruits, respectively (Additional file 12: Figure S8a-e). The root samples (Ro) expressed the most unigenes (49.80%), including 2653 upregulated and 2317 downregulated genes (Additional file 12: Figure S8f). The stems and fruits expressed the fewest unigenes (0.90 and 6.51%, respectively). To evaluate the functional properties of these organ-specific unigenes, KEGG enrichment was performed, and the significant pathways of each organ are listed in (Additional file 13: Table S5). Seventeen KEGG pathways were significantly enriched in root samples, including flavonoid biosynthesis, phenylpropanoid biosynthesis, terpenoid biosynthesis, alkaloid biosynthesis, and zeatin biosynthesis, which indicated that the most active biological synthesis was occurring in the roots of *Z. armatum*. Only two significant KEGG pathways (five unigenes) were observed in the stem samples. Additionally, 14 and 16 KEGG pathways were distinctively obtained in the LB and ML samples, respectively. In both cases, the greatest enrichment was observed for phenyl propanoid biosynthesis, glycerolipid metabolism, galactose metabolism, pentose and glucuronate interconversions, glycosphingolipid biosynthesis, and glycolysis and gluconeogenesis pathways, which supply the necessary substances for the growth and development of these tissues. However, unigenes involved in plant hormone signal transduction, flavonoid and terpenoid biosynthesis and metabolism were detected at significant levels in ML samples, which might contribute to the numbing taste and peppery flavour of the leaves of *Z. armatum*. Additionally, 18 significant KEGG pathways were identified in the Fr samples, most of which were enriched in terpenoid, alkaloid and flavonoid biosynthesis. These unigenes might be the main source of the special numbing taste of the fruits of *Z. armatum*.

### Identification and characterization of genes involved in terpenoid biosynthesis

**Fig. 2:**
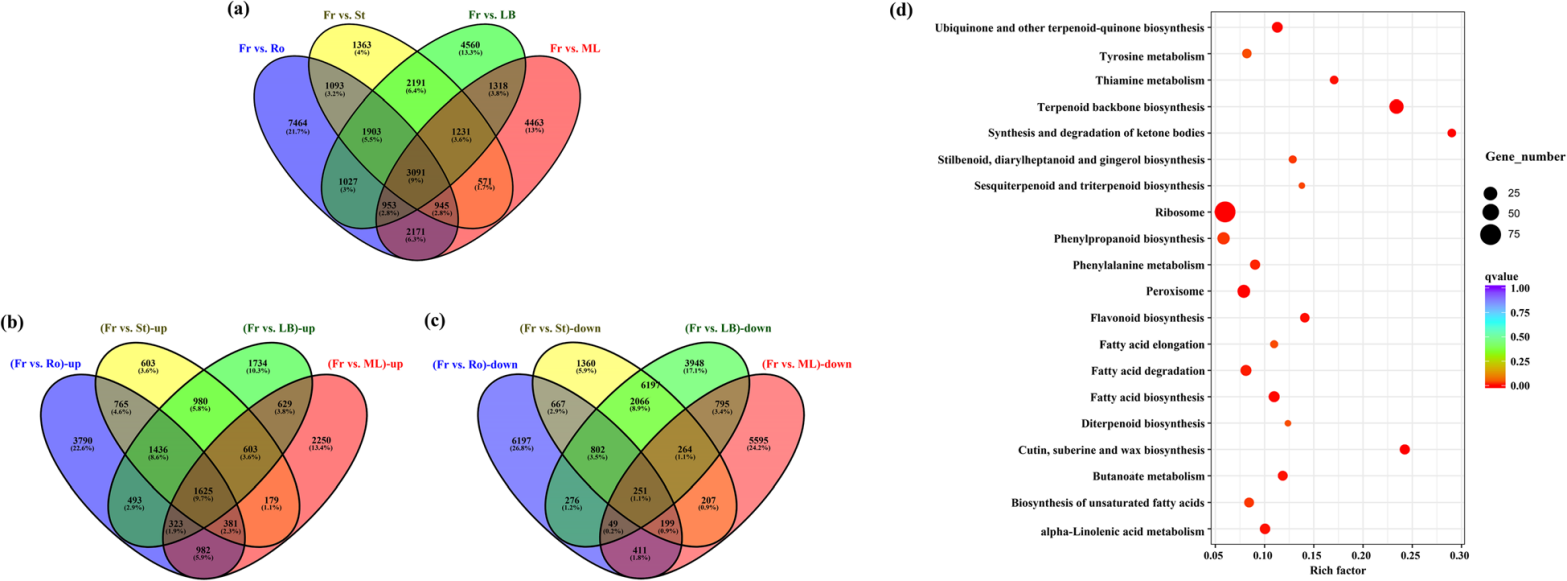
The DEGs analysis to screen the specific unigenes in fruit samples of *Z. armatum*. **a** the venny diagram of the DEGs detected in each comparison. **b** the venny diagram of the DEGs only up-regulated in fruit samples. **c** the venny diagram of the DEGs only down-regulated in fruit samples. **d** the statistics of KEGG pathway enrichment involved in DEGs only up-regulated in fruit samples. The Rich factor indicated the percentages of DEGs belong to the corresponding pathway. The sizes of bubble represent the number of DEGs in the corresponding pathway, and the colors of the bubble represent the enrichment q value of the corresponding pathway

The analysis of differentially expressed genes (DEGs) was carried out in the five major organs of *Z. armatum*. To comprehensively reveal the key genes associated with the special numbing taste in the fruits of *Z. armatum*, genes with a *p* value < 0.05 and a |log2(fold change)| >1 identified by EdgeR were regarded as DEGs in the comparisons of fruit samples and other organs (Fr vs. Ro, Fr vs. St, Fr vs. LB, and Fr vs. ML). As a result, a total of 3091 DEGs were co-detected in all four comparisons (Fig. 2a), 1625 of which were co-screened and found to be upregulated in all four comparisons (Fig. 2b), whereas 251 of which were co-identified and found to be downregulated in all four comparisons (Fig. 2c). The KEGG enrichment analysis showed that the downregulated DEGs were mainly related to plant hormone signal transduction and amino and sugar metabolism pathways (Additional file 14: Table S6). However, most of the upregulated DEGs were significantly enriched in terpenoid, alkaloid, flavonoid and fatty acid biosynthesis and metabolism (Fig. 2d, Additional file 14: Table S6). Thus, the following analysis was mainly focused on the upregulated unigenes to explore the genetic information associated with the special numbing taste in *Z. armatum*.

**Fig. 3:**
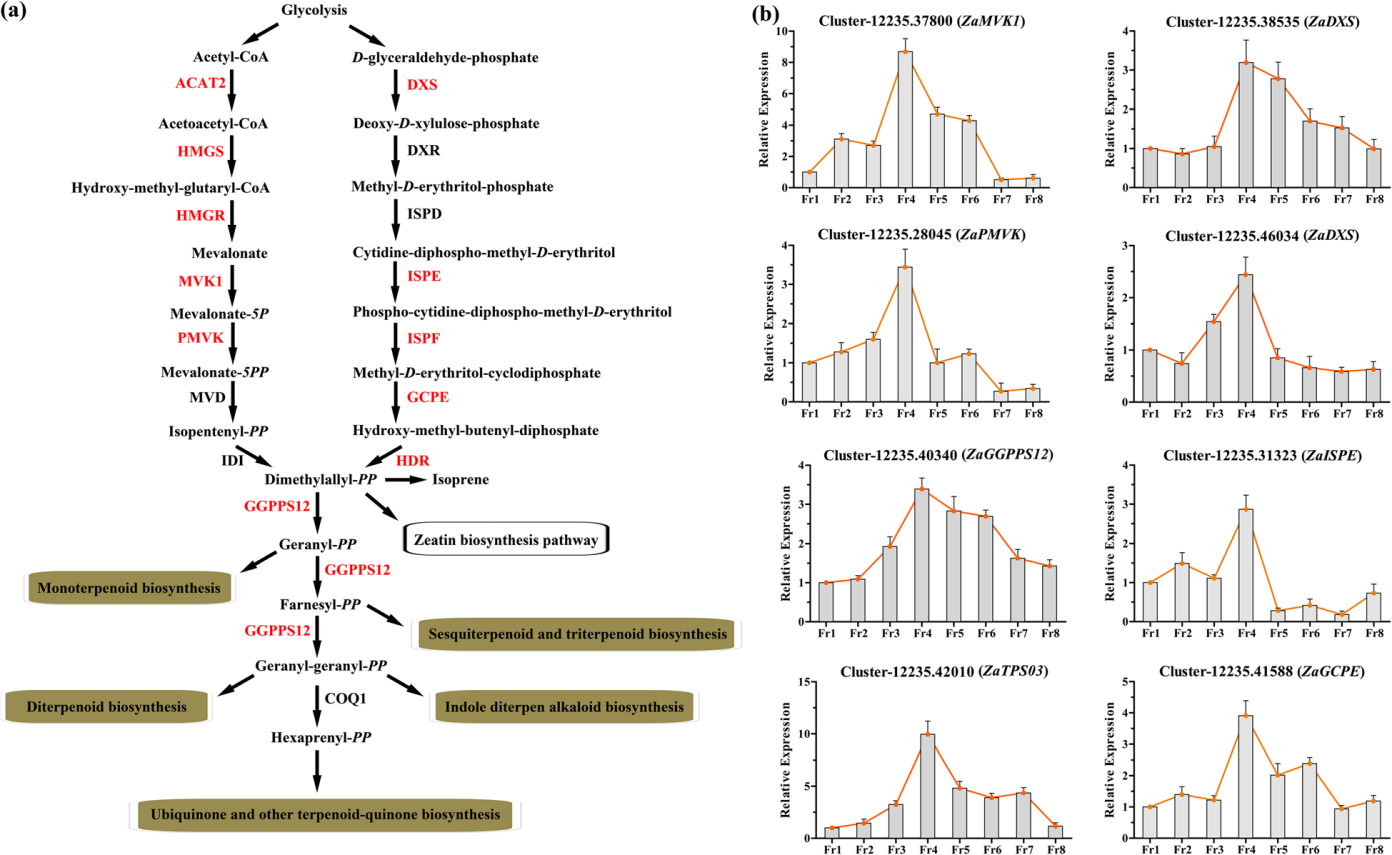
The identification of genes in the pathway of terpenoid biosynthesis based on the transcriptome of *Z. armatum*. **a** the regulatory cascade of terpenoid biosynthesis pathway. Red fonts indicates the homologous differential expressed genes significantly up-regulated in this study and they were abbreviated as follows: ACAT2, Acetoacetyl-CoA Thiolase 2; HMGS, Hydroxy-methyl-glutaryl-CoA Synthase; HMGR, Hydroxy-methyl-glutaryl-CoA Reductase; MVK1, Mevalonate Kinase 1; PMVK, Phosphomevalonate Kinase; DXS, Deoxy-D-Xylulose 5-Phosphate Synthase; ISPE, Diphosphocytidyl-methyl-D-erythritol Kinase; ISPF, Isoprenoid F; GCPE, Hydroxy-methylbut-enyl Diphosphate Synthase; HDR, Hydroxy-methylbut-enyl Diphosphate Reductase; GGPPS12, Geranylgeranyl diphosphate synthase 12. **b** the relative expression of the DEGs during fruit development and maturation process. The Fr1 was used as the control sample

In total, 53 DEGs were identified as candidate unigenes for 12 enzymes involved in terpenoid biosynthesis, and their expression values in the five major organs and TAIR10 annotations are shown in (Additional file 15: Table S7) and **(**Additional file 16: Table S8). These enzymes constituted two independent subpathways upstream of the terpenoid biosynthesis pathway (Fig. 3a), both of which utilize glycolysis to obtain the initial substrate for producing dimethylallyl diphosphate (dimethylallyl-*PP*). Then, dimethylallyl-*PP* is used to generate monoterpenoids, diterpenoids, triterpenoids, and other terpenoid compounds [14], which are the main aromatic substances involved in the special numbing taste in the fruits of *Z. armatum* [10]. In the present study, the results showed that almost all genes involved in terpenoid backbone biosynthesis were differentially expressed genes and detected at significant levels in the fruit samples (Fig. 3a).

To further characterize the functional properties of these DEGs, some of the DEGs were selected to perform qRT-PCR detection during fruit development and maturation. A total of eight fruit samples were collected to investigate these genes (Additional file 17: Figure S10): in Fr1, 5 days after fertilization, the fruit was green-yellowish with a smooth surface; in Fr2, 15 days after fertilization, the fruit was oval with some slightly concave and transparent speckling on the surface; in Fr3, 28 days after fertilization, the fruit was green and grew rapidly; in Fr4, 40 days after fertilization, the fruit was further expanded with obvious speckles; in Fr5, 50 days after fertilization, the fruit gradually stopped expanding, and inclusions began to accumulate within the speckles; in Fr6, 62 days after fertilization, the fruit was dark green, and significant speckles accumulated additional inclusions; in Fr7, 75 days after fertilization, the fruit gradually matured and exhibited many inclusions within speckles; and in Fr8, 85 days after fertilization, the fruit was completely mature, and the special numbing taste was fully developed.

Two unigenes, Cluster-12,235.37800 and Cluster-12,235.28045, were annotated to mevalonate kinase 1 (*MVK1*) and phosphomevalonate kinase (*PMVK*), respectively, which belong to the MVA pathway and play rate-determining roles in the production of mevalonate-*5PP*. The results showed that both of these unigenes were significantly upregulated in the Fr4 stage but showed lower expression in the preceding and the subsequent stages of fruit development and maturation, especially in the Fr7 and Fr8 stages. Cluster-12,235.38535 and Cluster-12,235.46034 were annotated to deoxy-*D*-xylulose phosphate synthase (*DXS*), which catalyses the initial step in the transformation of *D*-glyceraldehyde-phosphate into deoxy-*D*-xylulose-phosphate in the MEP pathway. The results showed that both of these unigenes were significantly upregulated in the Fr4 stage but downregulated in the preceding and subsequent stages of fruit development and maturation (Fig. 3b). Additionally, unigenes involved in two enzymes in the MEP pathway, Cluster-12,235.31323 (*ZaISPE*) and Cluster-12,235.41588 (*ZaGCPE*), presented similar expression patterns in the fruit development and maturation stages. Moreover, Cluster-12,235.40340, a key unigene annotated to geranyl-geranyl diphosphate synthase 12 (*GGPPS12*), which generates important substrates associated with the biosynthesis of various terpenoid compounds, presented gradual up-regulation from the Fr1 to Fr4 stages, whereas it was significantly downregulated in the Fr4 to Fr8 stages. A consistent result was that *ZaTPS03* (Cluster-12,235.42010) was exclusively upregulated in Fr4 samples of *Z. armatum*; this unigene is related to the catalysis of the production of (R)-limonene as well as other related compounds using geranyl-*PP* via the monoterpenoid biosynthesis process. These results indicated that the Fr4 stage could be the core period for the initiation of terpenoid compound biosynthesis and the accumulation of these compounds in the fruit development and maturation process of *Z. armatum*.

### Identification and characterization of genes involved in fatty acid biosynthesis

**Fig. 4:**
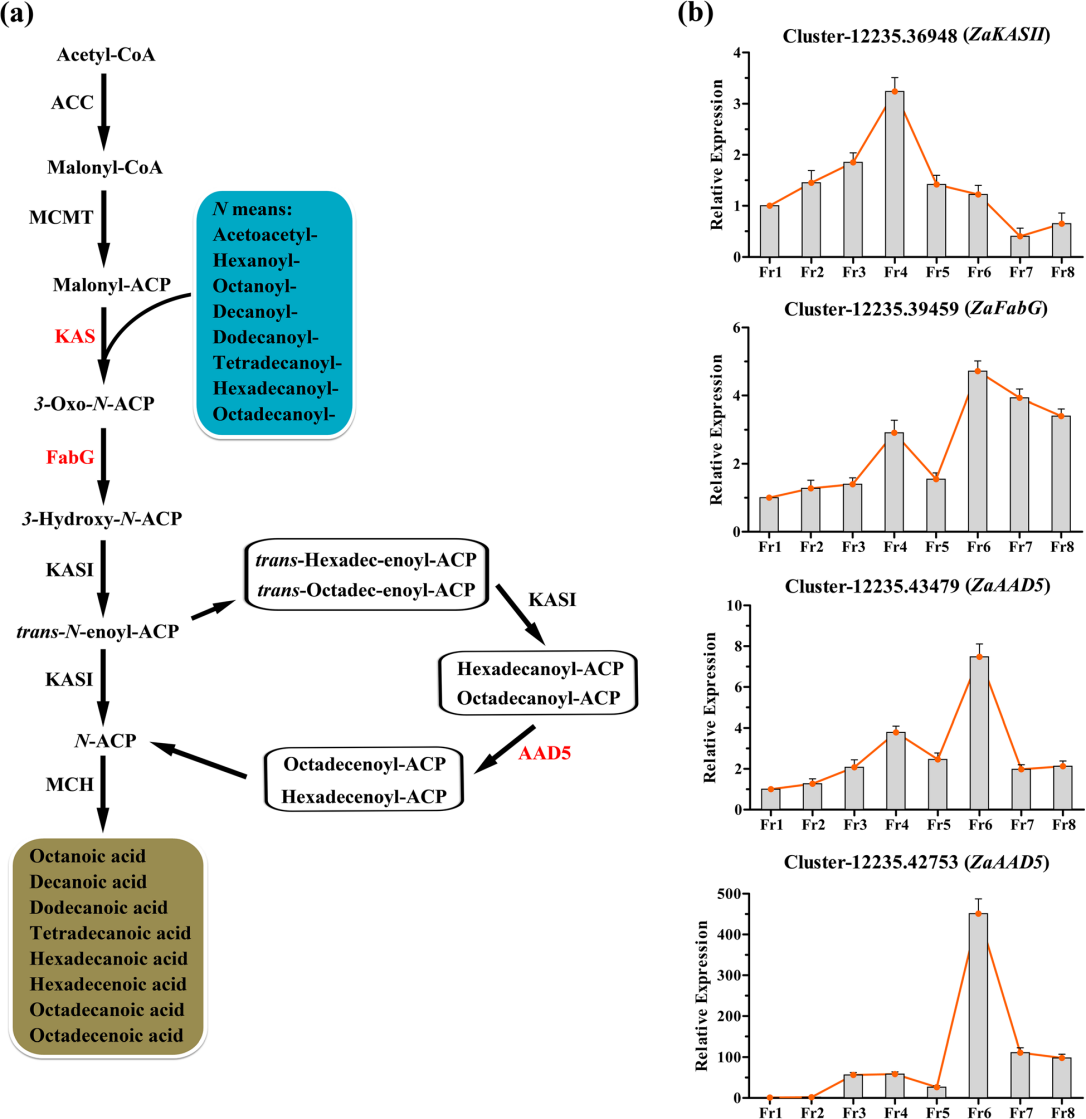
The identification of genes in the pathway of fatty acid biosynthesis based on the transcriptome of *Z. armatum*. **a** the regulatory cascade of fatty acid biosynthesis pathway. Red fonts indicates the homologous differential expressed genes significantly up-regulated in this study and they were abbreviated as follows and they were abbreviated as follows: ACP, acyl carrier protein; KASII, Ketoacyl-ACP Synthase II; FabG, Oxoacyl-acyl-carrier Protein Reductase; AAD5, ACP Desaturase 5. **b** the relative expression of the DEGs during fruit development and maturation process. The Fr1 was used as the control sample

Based on KEGG enrichment, a total of 20 DEGs were screened and annotated to fatty acid biosynthesis and elongation processes (Additional file 14: Table S6, Fig. 2d). The expression values of these candidate unigenes in the five major organs and their TAIR10 annotations are shown in (Additional file 15: Table S7) and (Additional file 16: Table S8). Moreover, all 20 DEGs were only associated with six subfamilies, including the ACP desaturase 5 (*AAD5*), acyl-activating enzyme 16 (*AAE16*), ketoacyl-ACP synthetase II (*KASII*), long-chain acyl-CoA synthase (*LACS*), ketoacyl-CoA synthase (*KCS*), and oxoacyl-acyl-carrier protein reductase (*FabG*) subfamilies, for which homologous *Arabidopsis* sequences were not identified in the TAIR10 protein database. Additionally, *KASII*, *FabG*, and *AAD5* were significantly involved in fatty acid biosynthesis pathways (Fig. 4a), whereas *LACS* and *KCS* were mainly associated with long-chain fatty acid biosynthetic and metabolic processes (Additional file 16: Table S8).

Furthermore, *ZaKASII* (Cluster-12,235.36948), a key synthetase related to the elongation of 16-carbon palmitoyl-ACP to produce 18-carbon stearoyl-ACP, was significantly upregulated in the Fr4 stage (Fig. 4b) but presented relatively low expression in the preceding and subsequent stages, especially in the fruit maturation process (Fr7-Fr8). However, Cluster-12,235.36948, annotatedto *FabG*, which reduces3-Oxo-*N*-ACP to form 3-hydroxy-*N*-ACP, was not only upregulated in the Fr4 stage but also showed higher expression in the Fr6-Fr8 stages. It is worth noting that two unigenes annotated to *ZaAAD5*, Cluster-12,235.43479 and Cluster-12,235.42753, showed similar patterns and were exclusively upregulated in the Fr6 stage. Both of these unigenes are involved in important reactions in the formation of long-chain unsaturated fatty acids.

### Identification and characterization of genes involved in fatty acid degradation

**Fig. 5:**
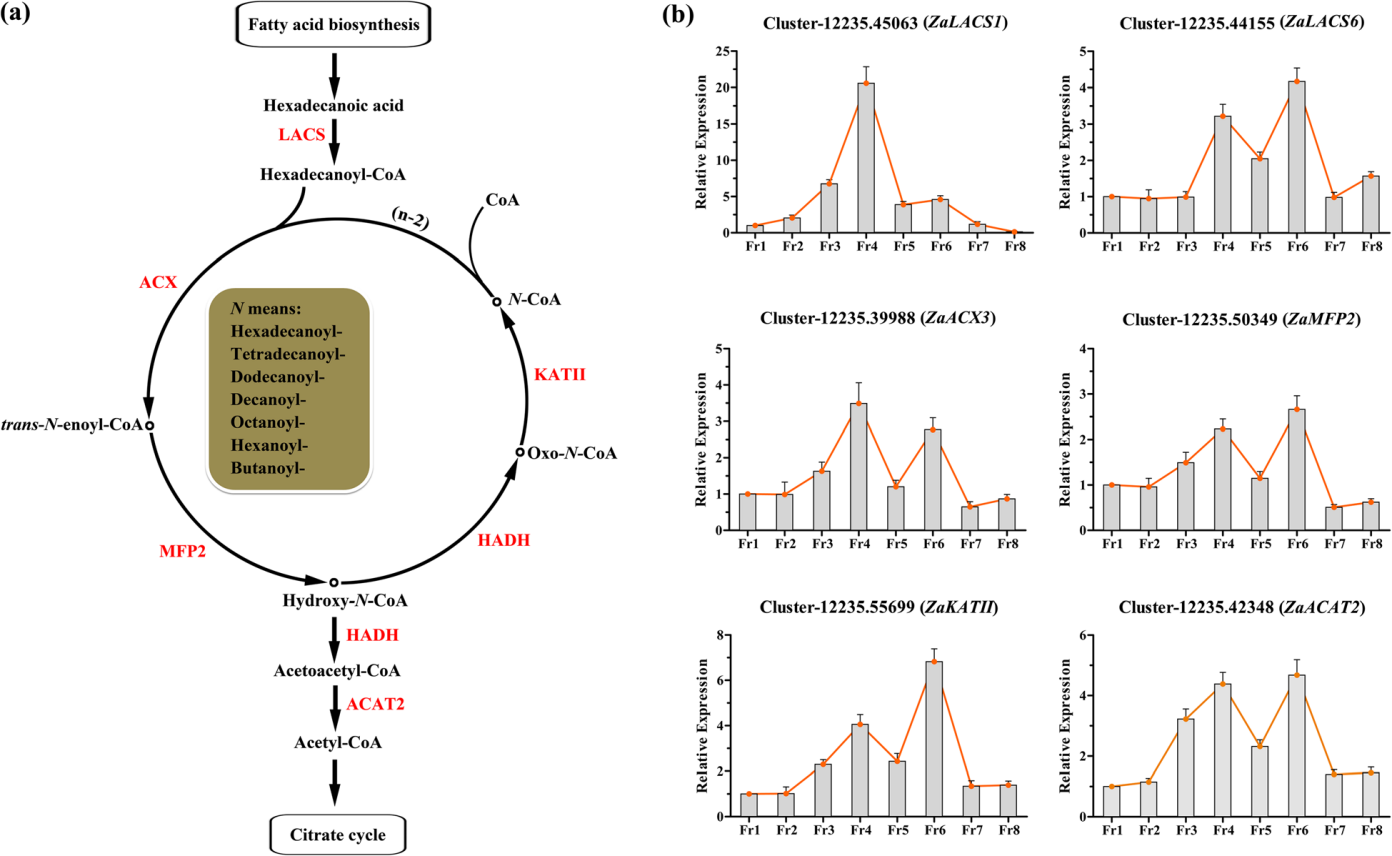
The identification of genes in the pathway of fatty acid degradation based on the transcriptome of *Z. armatum*. **a** the regulatory cascade of fatty acid degradation pathway. Red fonts indicates the homologous differential expressed genes significantly up-regulated in this study and they were abbreviated as follows: LACS, Long-chain Acyl-CoA Synthetase; ACX3, Acyl-CoA Oxidase 3; MFP2, Multi-functional Protein 2; HADH, Hydroxyacyl-CoA Dehydrogenase; KATII, Ketoacyl-CoA Thiolase II; ACAT2, Acetoacetyl-CoA Thiolase 2. **b** the relative expression of the DEGs during fruit development and maturation process. The Fr1 was used as the control sample

A total of 168 unigenes were annotated to fatty acid degradation pathways in the transcriptome database analysed in the present study, and 14 DEGs were screened and found to be significantly upregulated in fruit samples according to KEGG enrichment analysis (Additional file 14:Table S6, Fig. 2d). The expression values of these candidate DEGs in the five major organs and their TAIR10 annotations are also listed in (Additional file 15: Table S7) and **(**Additional file 16: Table S8). Interestingly, the major pathway of fatty acid degradation was beta-oxidation catabolism, and the main enzymes involved in each step were identified in our DEG profiles, which included long-chain acyl-CoA synthase (*LACS*), acyl-CoA oxidase (*ACX*), multifunctional protein 2 (*MFP2*), hydroxyacyl-CoA dehydrogenase (*HADH*), ketoacyl-CoA thiolase (*KAT*) and acetoacetyl-CoA thiolase 2 (*ACAT2*) (Fig. 5a).

The two *LACS* genes were investigated in the present study, whose encoded proteins catalyse the initial reactions of the fatty acid degradation process. Cluster-12,235.42753, annotated to *ZaLACS1*, was only very significantly upregulated in the Fr4 stage (Fig. 5b), whereas Cluster-12,235.42753 (*ZaLACS6*) was not only upregulated in the Fr4 stage but was also highly expressed in the Fr6 stage. Similar results were obtained for *ZaACX3* (Cluster-12,235.39988) and *ZaMFP2* (Cluster-12,235.50349), which catalyse the first and second reactions of fatty acid *β*-oxidation, respectively, and presented two peaks of upregulation (Fr4 and Fr6). Additionally, their downstream oxidase, *ZaKATII* (Cluster-12,235.55699), which generates and releases CoA, was distinctively upregulated in the Fr4 and Fr6 stages. Additionally, *ZaACAT2* (Cluster-12,235.42348), which is involved in a key step in the transformation of acetoacetyl-CoA into acetyl-CoA, was still highly expressed in the Fr4 and Fr6 stages of *Z. armatum*.

### qRT-PCR validation

To experimentally confirm the RNA-seq data, eight DEGs (with 21 terms) detected in Frvs. Ro, Frvs. St, Frvs.LB, Frvs.ML, LB*vs.* Ro, ML*vs.*LB, ML*vs.* Ro, and ML*vs.* St were tested by qRT-PCR (Additional file 18: Table S9). These genes were randomly selected based on their high fold changes indicating their crucial functions identified in this study. The results for all of these genes were consistent and showed the same trend of up- or downregulation between the qRT-PCR and the RNA-seq platform (Additional file 19: Figure S11). The correlation coefficient for the two expression measurements was 0.8059 between these 24 terms (*R*^2^ = 0.8059). In summary, the results suggested that our transcriptome data accurately reflected the expression patterns of most genes in *Z. armatum*.

## Discussion

In the present study, the five major organs of *Z. armatum* were collected to construct a comprehensive transcriptome dataset with abundant genetic information using the Illumina HiSeq™ 4000 platform in *Z. armatum*. All the unigenes were subjected to BLASTx searches to characterize their gene functions against five public databases, and the species distribution of the NR BLASTx matches showed that *Z. armatum* was presents a close relationship to *Citrus* species. In fact, both *Citrus* and *Zanthoxylum* belong to Rutaceae [25]. The consistent results indicated that *Zanthoxylum* is a relatively recent species that is predicted to have diverged from *Citrus* 36.5–37.7 million years ago [1, 26].

Additionally, the present study showed that at least 9–12 repeats were detectable for monomer nucleotide SSRs, whereas the di- to hexanucleotide SSRs were explored mostly in the context of 5 to 8 repeats. These results indicated that more repeats were likely to be detected for repeat types with fewer nucleotides, which is consistent with reported studies in various plants [27], and the types of SSRs are affected by the selective pressure on different genes [28]. In summary, a large number of molecular markers were detected in the present study, indicating that high variation might exist in *Z. armatum.* In a previous study, 11 pairs of primers were used to clearly distinguish *Zanthoxylum bungeanum* (red Sichuan pepper) from *Zanthoxylum armatum* (green Sichuan pepper), even differentiating various cultivars [1]. The molecular markers screened in the present study provide rich information and could contribute to the improvement of a molecular marker database that can be used for revealing high polymorphism and multiple alleles and for marker-assisted selection breeding in *Zanthoxylum* species.

Additionally, this study identified a large number of organ-specific unigenes that were significantly associated with development and other biological processes. All the KEGG enrichments for each organ were consistent with previous studies showing that components responsible for the special numbing taste, including volatile oils, alkaloids, terpenoids, acid amide phenol components and so on [6, 7], accumulate at high levels in the fruits, leaves and roots of *Z*. *armatum* [8]. These results could also reveal many candidates and underlying specific promoters related to the special numbing taste, and further research could focus on confirming their molecular functions in model plants (such as *Arabidopsis thaliana*) and *Z. armatum* to provide more genetic information about these genes.

The fruit of *Z. armatum* is the main organ in which numbing taste-related compounds accumulate [8]. Thus, the present study screened DEGs related to terpenoid biosynthesis and fatty acid biosynthesis in the comparisons of fruit samples with other organs (Fr *vs*. Ro, Fr *vs*. St, Fr *vs*. LB, and Fr *vs*. ML). This approach identified 53 DEGs related to 12 enzymes involved in the terpenoid biosynthesis process, covering almost all of the important enzymes associated with terpenoid backbone biosynthesis. These results indicated that the fruit is a key location of the synthesis of terpenoid compounds in *Z. armatum*. Therefore, the eight samples were harvested to further identify their expression patterns in fruit development and maturation stages. Eight important candidate genes were selected for qRT-PCR analysis, including *ZaDXS*, *ZaISPE*, *ZaGCPE*, *ZaMVK1*, *ZaPMVK*, *ZaTPS03* and *ZaGGPPS12*. Two unigenes, Cluster-12,235.38535 and Cluster-12,235.46034, were annotated to deoxy-*D*-xylulose phosphate synthase (*DXS*), which is recognized as a rate-limiting enzyme that catalyses the first step in the MEP pathway [29]. The functions of *DXS* have been characterized in multiple plants. The overexpression of *LiDXS* from *Lilium* ‘Siberia’ substantially increases the diterpene content of tobacco [29]. Other studies have also reported that *DXS* plays a crucial role in the biosynthesis of the monoterpene precursor [30–33]. Additionally, *GbMVK* is highly expressed in the leaves, roots and stems of *Ginkgo biloba*, which is related to the production of the main active substance in terpene trilactone biosynthesis [34]. In the classical MVA pathway, PMVK can catalyse the transformation of mevalonate-*5P* into mevalonate-*5PP*, which is subsequently subjected to decarboxylation catalysed by mevalonate 5-diphosphate decarboxylase [14]. Recently, Henry et al. found that PMVK is a key regulatory hub in controlling the flux through the plant MVA pathway [14]. Moreover, Cluster-12,235.40340 was annotated to geranyl-geranyl diphosphate synthase 12 (*GGPPS12*). *GGPPS* is composed of an inactive small subunit and an active large subunit and generates important substrates associated with the biosynthesis of monoterpene compounds in various plants [35–37]. *ZaTPS03* (Cluster-12,235.42010) was also identified in this study, which related to a typical monoterpene synthase that catalyses the transformation GPP into limonene as well as other related compounds [22]. Previous studies have shown that *TPS* family members are the main contributors to the production of monoterpenes and sesquiterpenes in the plant kingdom [36]. In the present study, it was notable that all eight of these genes were significantly upregulated in the Fr4 stage but downregulated in the preceding and subsequent stages of fruit development and maturation. A consistent result was that terpenoids predominantly accumulated in the mid-developmental period of the fruit maturation process in *Zanthoxylum bungeanum* [22]. Similar expression patterns can be detected during fruit development in finger citron [38], tomato [39] and other species [40]. Considering the results of previous work and the present study together, we suggest that forty days after fertilization could be a core period for the initiation of the biosynthesis of terpenoid compounds and their accumulation in the fruit development and maturation process of *Z. armatum*. In further studies, a strategic approach will be to measure the concentrations of various terpenoid compounds in fruit differentiation stages via metabolomics analysis coupled with high-performance liquid chromatography (HPLC) fingerprinting [5, 41], and the functions of crucial genes could also be evaluated with transgenic methods to verify the present results in depth.

Finally, this study also screened 34 DEGs associated with fatty acid biosynthesis, elongation and degradation pathways from the 347 background unigenes in the present transcriptome dataset (Additional file 14: Table S6). According to the RNA-seq analysis, all of these DEGs were exclusively upregulated in fruit samples. Similarly, ten candidate genes were investigated for their expression patterns in fruit development and maturation stages. The function of *KASII* has been fully elucidatedin many different higher plants, and its main role is to catalyse the elongation of palmitoyl-ACP [42]. Four unigenes were annotated to *FabG* in the KEGG pathways, which can reduce 3-Oxo-*N*-ACP to form 3-hydroxy-*N*-ACP [43]. However, the genetic information and gene names of these unigenes were not annotated in the TAIR10 database. The family members of *AAD5*are responsible for long-chain unsaturated fatty acid biosynthesis and are predominantly expressed throughout seed developmental stages. The *aad5* mutation of *Arabidopsis* can cause a significant reduction in the concentration of C18:1 fatty acids [44]. The present study showed that *ZaKASII* was significantly upregulated in the Fr4 stage, whereas both *ZaAAD5* genes were exclusively upregulated in the Fr6 stage. Additionally, *ZaFabG* showed two expression peaks, not only being upregulated in the Fr4 stage but also showing high expression in the Fr6-Fr8 stages. DEGs related to the fatty acid degradation pathway were also identified in fruit development and maturation stages, including *ZaLACS*, *ZaACX3*, *ZaKATII* and *ZaACAT2*. Multiple studies have explored the genetic functions of these genes involved in fatty acid accumulation and degradation in *Arabidopsis thaliana* and other plants [45–48]. Additionally, *ACAT2* is typically related to terpenoid biosynthesis in the mevalonate pathway, where it can reversibly catalyse formation of acetyl-CoA from acetoacetyl-CoA [12]. It is worth noting that almost all of these genes exhibited two upregulation peaks in the Fr4 and Fr6 stages, except for *ZaLACS1,* which was only very significantly upregulated in the Fr4 stage. These results were consistent with the expression patterns of the genes in fatty acid biosynthesis pathways detected in this study (Figs. 4 and 5). In a previous study, genes related to fatty acid biosynthesis were grouped into two categories and detected in the early or later stages of seed development in tree peony [49]. Similarly, the coordinated regulation of multiple genes has been shown to promote the high accumulation of long-chain unsaturated fatty acids in the seeds of tea oil camellia [50], and consistent results have been obtained in *Brassica napus* [51, 52], *Glycine max* [53], and *Linum usitatissimum* [54]. Above all, the present results indicated that 40 days after fertilization could be a key point at which the first few steps of fatty acid biosynthesis are promoted in *Z. armatum*, and the subsequent reactions could be catalysed and completed in the stage occurring 62 days after fertilization, especially for long-chain fatty acid biosynthesis.

The present study only investigated the expression patterns of certain crucial candidate genes involved in terpenoid compound and fatty acid biosynthesis and metabolism. Further study could focus on identifying the relative expression levels of more genes related to the pathways of alkaloids, coumarins, acid amide phenol components and so on from the present transcriptome dataset. Additionally, a further study could be devoted to confirming the molecular functions of these genes via the overexpression of the genes driven by the 35S promoter and/or their silencing by hairpin RNAs in transgenic *Z. armatum* plants, which might reveal more genetic information underlying the regulatory mechanism associated with the special aromatic taste of *Z. armatum*.

## Conclusions

The present study achieved de novo transcriptome assembly for five major organs in *Z. armatum*. A total of 111,318 nonredundant unigenes were generated, with an average length of 1014 bp, 73,426 of which could be functionally annotated in at least one database. Additionally, a large number of SSRs were obtained to improve our understanding of the phylogeny and genetics of some important traits of *Z. armatum*. The organ-specific unigenes of the five major samples were screened and annotated via GO and KEGG enrichment analysis. Additionally, 1876 unigenes were found to be exclusively up- or downregulated in fruit samples, 53 of which were identified as candidate unigenes for 12 enzymes involved in terpenoid biosynthesis, 34 of which were significantly annotated to fatty acid biosynthesis, elongation and degradation pathways. Furthermore, qRT-PCR detection was conducted in eight fruit development and maturation stages. Forty days after fertilization could be a crucial period for the initiation of the biosynthesis of terpenoid compounds and their accumulation in the fruit development and maturation process of *Z. armatum*. However, the Fr4 stage could be a key point at which the upstream steps of fatty acid biosynthesis are promoted in *Z. armatum*, and the subsequent reactions could be significantly activated in the Fr6 stage, especially long-chain unsaturated fatty acid biosynthesis.

The present dataset provides a reference transcriptome for the genomic database of *Z. armatum* for future studies. To the best of our knowledge, this study is the first to perform a comprehensive analysis revealing the underlying genes related to the special numbing taste in *Z. armatum*. The assembled transcriptome profiles expand the available genetic information for *Z. armatum* and will contribute to gene functional studies, which will aid in the engineering of high-quality varieties of *Z. armatum*.

## Methods

### Plant material collection

Three-year-old *Z. armatum* planted at a forestry trial base of Sichuan Agricultural University (30.60°N, 103.65°E) in Chengdu City, Sichuan Province, China, was selected as the experimental material in the present study. The material has been authenticated by the Forestry Variety Certification Committee of Sichuan Province, and the deposition number is SV-ZA-002-2018. This improved variety, *Z. armatum* ‘Hanyuan putaoqing’, was bred by our coauthor Professor Wei Gong (from Sichuan Agricultural University). The trial site is located in a subtropical humid monsoon climate area, with an annual average sunshine of > 1160 h, mean annual rainfall of 1012 mm, and a mean annual temperature of 15.9 °C. Five organs, including the roots (Ro), stems (St), leaf buds (LB), mature leaves (ML) and fruits (Fr), were harvested for RNA-seq analysis in May 2019. These samples were collected at approximately 60 days after fertilization, when the fruits had reached full size and were rapidly accumulating aromatic compounds and fatty acids. Additionally, the fruit samples were harvested approximately every 10 days during fruit development and maturation from April to July (Additional file 17: Figure S10). In total, eight samples (Fr1 to Fr8) were obtained to investigate the genes associated with terpenoid compound and fatty acid metabolism in the present study. Each sample consisted of material collected from three individual plants, which was mixed together as a biological replicate, and three independent biological replicates were performed. All samples were flash frozen in liquid nitrogen and stored at − 80 °C until being used for RNA extraction.

### Total RNA extraction and transcriptome sequencing analysis

The total RNA of each sample was extracted separately using the Magen Plant RNA Kit (HiPure HP Plant RNA Mini Kit, Guangzhou) following the instructions of the manufacturer. The quality of the total RNA was investigated by using agarose gel electrophoresis and a Nanodrop 2100 spectrophotometer (Agilent, USA). The 15 cDNA libraries in this study (Ro_1, Ro_2, Ro_3, St_1, St_2, St_3, LB_1, LB_2, LB_3, ML_1, ML_2, ML_3, Fr_1, Fr_2, and Fr_3) were constructed and sequenced using the Illumina HiSeq™ 4000 (Illumina, USA) platform at Novegene Bioinformatics Technology Co. Ltd. (Beijing, China). Sequence adaptors, reads with more than 10% N bases and low-quality reads (Q phred ≤20 for > 50% read) were removed to obtain clean data according to the method described by Lv et al. [55]. After quality control procedures, the unigenes were de novo assembled using Trinity software [56]. All of the transcriptome data were used as a reference dataset to calculate the read count of each unigene among the samples and converted to the expected number of fragments per kilobase of transcript sequence per million base pairs sequenced (FPKM) as described by Trapnell et al. [57].

### Gene functional annotation and DEG analysis

To obtain comprehensive functional information, all unigenes were aligned by BLASTx to the NCBI nonredundant protein sequence (NR), NCBI nucleotide sequence (NT), Protein family (Pfam), KEGG Ortholog (KO) and euKaryotic Ortholog Groups (KOG) databases with a threshold E-value of 10^− 5^. Additionally, the functional annotation and classification of all the unigenes were also performed according to the Gene Ontology database (GO, http://www.geneontology.org/) and the Kyoto Encyclopedia of Genes and Genomes database (KEGG, https://www.kegg.jp/).

The differential expression analysis of the samples with three biological replications was conducted using edgeR [58]. The fold change (FC) is the gene expression difference between different samples. The organ-specific unigenes of the five major samples were screened according to a *q* value < 0.05 and |log2(fold change)| >5, and the differentially expressed genes (DEGs) between different samples were identified according to a *q* value < 0.05 and |log2(fold change)| >1. All of the up- or downregulated genes described in the present study are illustrated by the first comparison component. GOSeq and KOBAS software were used to estimate the statistical enrichment of DEGs in GO terms and KEGG pathways, respectively [59, 60]. A corrected *p*-value of 0.05 was set as the threshold for the significant enrichment of GO terms and KEGG pathways. BLASTx alignments were performed for the DEGs in the TAIR 10 database (https://www.arabidopsis.org/). The volcano plot and heat maps of the DEGs identified in this study were drawn with R software (R-2.15.3-win) according to the procedure described by Silva et al. [61].

### Identification of simple sequence repeats

The Perl script MISA was used to identify the simple sequence repeats (SSRs) according to the procedure described by Wang et al. [62]. Similar criteria to those employed in previous studies were used for the screening of high-quality SSRs in the five different samples collected in this study [20, 27].

### Quantitative real-time PCR analysis

To validate the transcriptome results, a total of eight DEGs (21 terms) detected in Fr*vs.* Ro, Fr*vs.* St, Fr*vs.*LB, Fr*vs.*ML, LB*vs.* Ro, ML*vs.*LB, ML*vs.* Ro, and ML*vs.* St were selected for quantitative real-time PCR (qRT-PCR) analysis using the same plant materials employed for RNA sequencing. These genes were randomly selected based on their high fold changes and crucial functions identified in this study (Additional file 18: Table S9). Three biological replicates were carried out for the different organ samples, with three technological replications for each gene. Moreover, the DEGs involved in terpenoid compound and fatty acid metabolism were selected for qRT-PCR analysis using eight samples related to fruit development and maturation. cDNA synthesis was performed using the PrimeScript® II First Strand cDNA Synthesis Kit (TaKaRa, Japan). The specific primers were designed with Primer Premier 5.0, and the amplified PCR products varied from 80 to 300 bp in length (Additional file 20: Table S10). qRT–PCR was performed in a Bio-Rad CFX96™ system (Bio-Rad, America) with SYBR Premix Ex Taq™ II (TaKaRa, Japan). *ZaUBQ* was selected as an internal control according to a previous study [2]. The 2^-ΔΔCt^ method was used to calculate the relative expression levels of the genes in different samples [63]. Fr1 (Fruit 1) was used as a control sample to estimate the relative expression of the DEGs associated with terpenoid compounds and fatty acid metabolism during the fruit development and maturation process in this study. GraphPad Prism 5 was used to draw the figures [64].

## Supplementary information

**Additional file 1: Table S1.** The quality of RNA extration.

**Additional file 2: Table S2.** Overview of the sequencing data of *Zanthoxylum armatum* transcriptome.

**Additional file 3: Figure S1.** The length distribution of transcripts (a) and unigenes (b). The inserts show the frequency distribution of the transcript length (a) and of the unigene length (b).

**Additional file 4: Table S3.** The summary of SSR in the total of unigenes.

**Additional file 5: Figure S2.** The overview of *Zanthoxylum armatum* transcriptome assembly and the characteristics of the homology search of unigenes. (a) e-value distributions of the best BLAST hits for each unigene against the NR database. (b) similarity distribution of the best BLAST hits for each unigene against the NR database. (c) venn diagram showing the BLAST searches of the *Zanthoxylum armatum* transcriptome against the five public databases. (d) species distribution of the best BLAST hit for each unigene against the NR database.

**Additional file 6: Figure S3.** Gene ontology distributions for the transcriptome of five major samples in *Z. armatum*. Main functional categories of the transcriptome related to plant physiology of the biological process, cellular component, and molecular function. The abscissas show the number of unigenes, and one unigene may be associated with different GO terms.

**Additional file 7 Figure S4.** The KEGG pathway for the transcriptome of five major samples in *Z. armatum*. The unigenes were divided into five clusters according to KEGG metabolism pathways, A: Cellular processes, B: Environmental information processing, C: Genetic information processing, D: Metabolism, E: Organismal systems.

**Additional file 8: Table S4.** KEGG annotation of nonredundant unigenes in *Zanthoxylum armatum*.

**Additional file 9: Figure S5.** Heat map representation and hierarchical clustering of putative genes involved in fatty acid metabolism pathways.

**Additional file 10: Figure S6.** Heat map representation and hierarchical clustering of putative genes involved in terpenoid compounds biosynthesis pathways.

**Additional file 11: Figure S7.** The pearson correlation coefficient (*R*^*2*^) was used to estimate the difference between the replicates of each tissue. The number between these two samples is given in the plot. The color represents R value, which shows high correlation in blue between two samples, while low correlation in white.

**Additional file 12: Figure S8.** The volcano plot of differential expressed genes (DEGs) associated with organ-specific unigenes. (a)-(e) represent the organ-specific unigenes in Ro, St, LB, ML and Fr, respectively. The abscissa shown the fold change of DEGs in each comparison and the ordinate shows the significance of DEGs, *q* < 0.05. The red dot was up regulation, the blue dot was down regulation, and the green dot was not significant difference. (f) was the percentage of unigenes expressed in each organ.

**Additional file 13: Table S5.** The significant pathways of KEGG enrichment related to organ-specific unigenes in *Zanthoxylum armatum*.

**Additional file 14: Table S6.** The significant paythways of KEGG enrichment related to the unigenes only up- or down- regulation in fruit samples of *Zanthoxylum armatum*.

**Additional file 15: Table S7.** The FPKM of the unigenes detected in the significant KEGG enrichment pathways of only up-regulation in fruit samples of *Zanthoxylum armatum*.

**Additional file 16: Table S8.** The TAIR annotation of the DEGs associated with terpenoid compounds and fatty acids biosynthesis and metabolism in fruit samples of *Zanthoxylum armatum*.

**Additional file 17: Figure S10.** The samples involved in fruit development and maturation in *Z. armatum*. (a)-(h) represent the samples collected from Fr1 to Fr8, respectively. All the fruit samples were harvested from April to July 2019. The bar is 2 mm.

**Additional file 18: Table S9.** The RNA-seq data of the DEGs selected for qRT-PCR validation.

**Additional file 19: Figure S11.** The correlation between qRT-PCR and RNA-seq data. Correlation between qRT-PCR and RNA-seq data of eight DEGs (21 terms) selected in Fr*vs.* Ro, Fr*vs.* St, Fr*vs.*LB, Fr*vs.*ML, LB*vs.* Ro, ML*vs.*LB, ML*vs.* Ro, and ML*vs.* St, including 6 down-regulation and 15 up-regulation. Pearson correlation coefficient = 0.8059 (*p* < 0.05).

**Additional file 20: Table S10.** The primers of qRT-PCR used for validation of expression trend in *Zanthoxylum armatum*.

## Data Availability

The data supporting the results presented in this article are included as additional files. All RNA-seq data in this study are archived in NCBI GEO database with the accession number GSE142491.

## Abbreviations

AAD5: ACP desaturase 5
ACAT2: Acetoacetyl-CoA thiolase 2
ACP: acyl carrier protein
ACX3: Acyl-CoA oxidase 3
DEG: Differential expressed genes
DXS: Deoxy-D-xylulose 5-phosphate synthase
FabG: Oxoacyl-acyl-carrier protein reductase
FC: Fold change
GCPE: Hydroxy-methylbut-enyl diphosphate synthase
GGPPS12: Geranylgeranyl diphosphate synthase
GO: Gene ontology
HADH: Hydroxyacyl-CoA dehydrogenase
HDR: Hydroxy-methylbut-enyl diphosphate reductase
HMGR: Hydroxy-methyl-glutaryl-CoA reductase
HMGS: Hydroxy-methyl-glutaryl-CoA synthase
ISPE: Diphosphocytidyl-methyl-D-erythritol kinase
ISPF: Isoprenoid F
KASII: Ketoacyl-ACP synthase II
KATII: Ketoacyl-CoA thiolase II
KEGG: Kyoto encyclopedia of genes and genomes
LACS: Long-chain acyl-CoA synthetase
MFP2: Multi-functional protein 2
MVK1: Mevalonate kinase 1
NCBI: National center for biotechnology information
PMVK: Phosphomevalonate Kinase
SNP: Single nucleotide polymorphism
SSR: Simple sequence repeats
TAIR: The Arabidopsis information resource
*Z*. *armatum*: *Zanthoxylum armatum*
