# Supplementary figures and images for: De novo transcriptome assembly for the five major organs of Zanthoxylum armatum and the identification of genes involved in terpenoid compound and fatty acid metabolism
Source: BMC Genomics. 2020 Jan 28;21:81. doi: 10.1186/s12864-020-6521-4 (PMC6986037; doi:10.1186/s12864-020-6521-4)

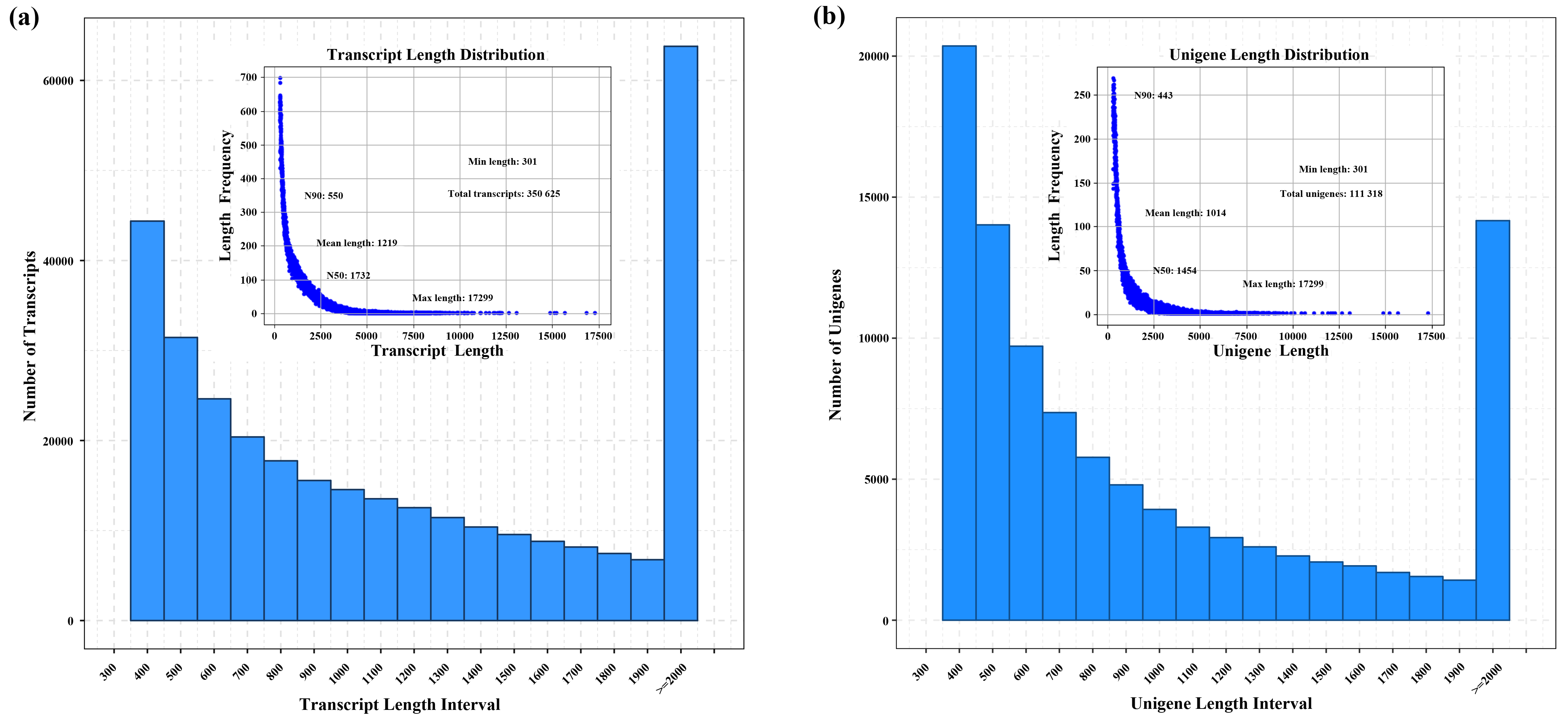

Supplement: Supplementary file 3 — Additional file 3: Figure S1. The length distribution of transcripts (a) and unigenes (b). The inserts show the frequency distribution of the transcript length (a) and of the unigene length (b). [file 12864_2020_6521_MOESM3_ESM.png]

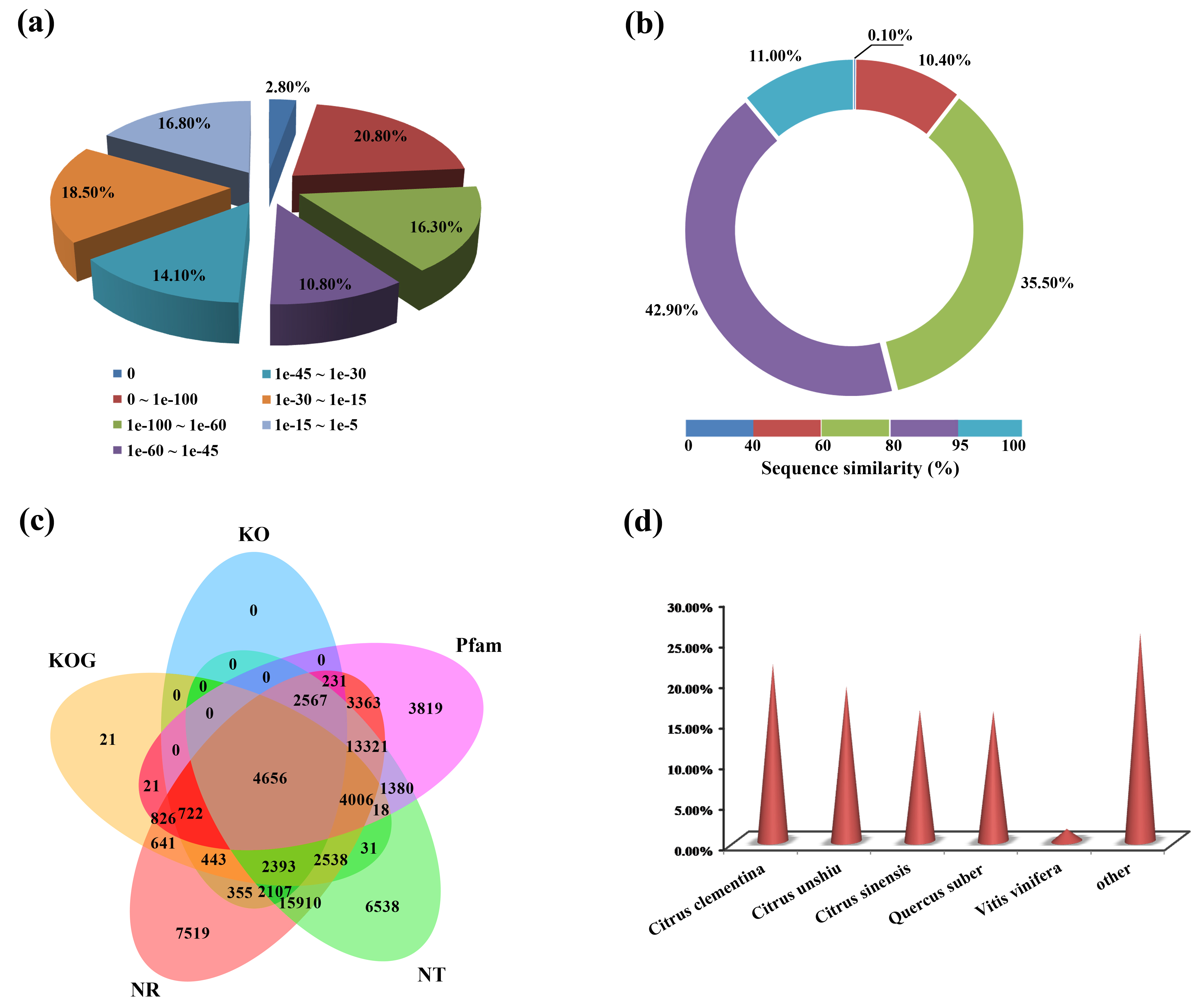

Supplement: Supplementary file 5 — Additional file 5: Figure S2. The overview of Zanthoxylum armatum transcriptome assembly and the characteristics of the homology search of unigenes. (a) e-value distributions of the best BLAST hits for each unigene against the NR database. (b) similarity distribution of the best BLAST hits for each unigene against the NR database. (c) venn diagram showing the BLAST searches of the Zanthoxylum armatum transcriptome against the five public databases. (d) species distribution of the best BLAST hit for each unigene against the NR database. [file 12864_2020_6521_MOESM5_ESM.jpg]

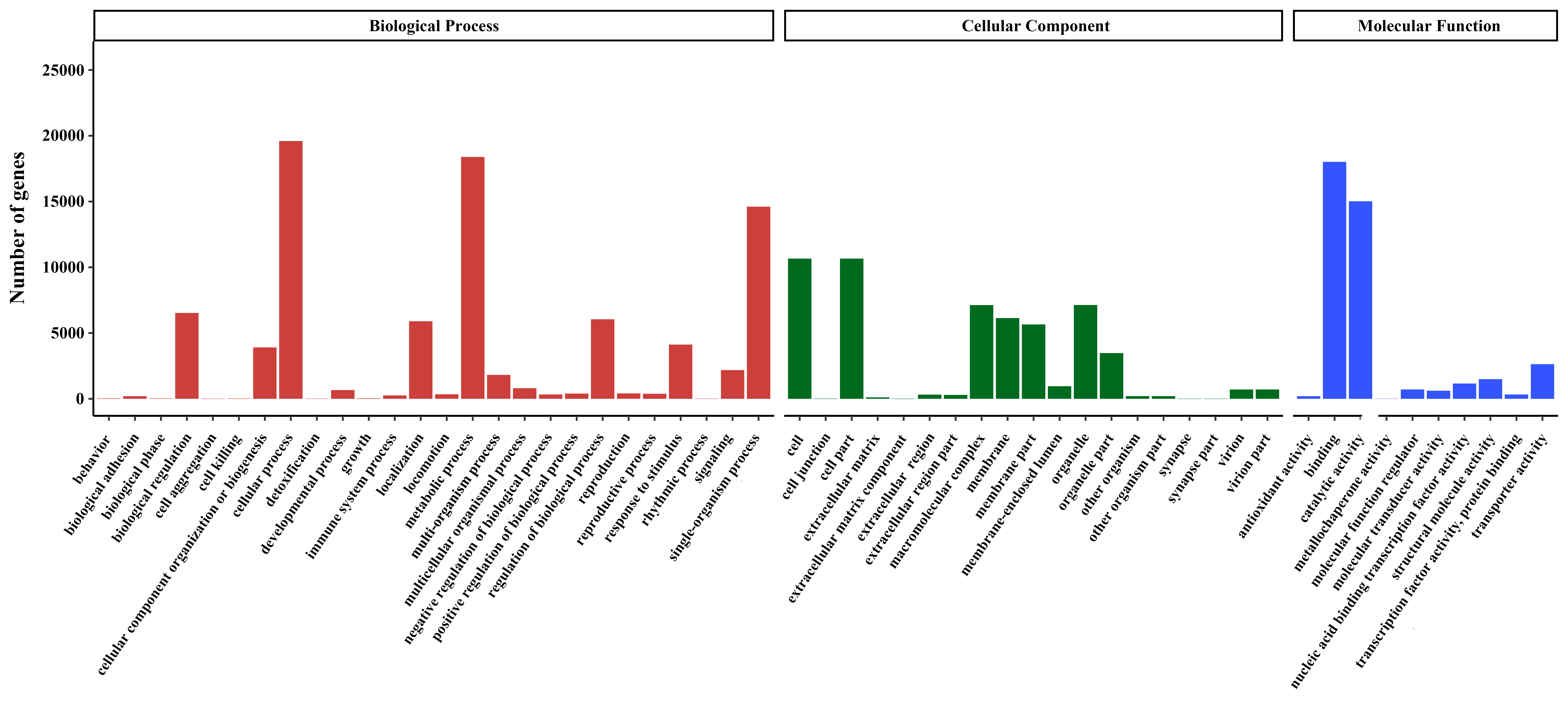

Supplement: Supplementary file 6 — Additional file 6: Figure S3. Gene ontology distributions for the transcriptome of five major samples in Z. armatum. Main functional categories of the transcriptome related to plant physiology of the biological process, cellular component, and molecular function. The abscissas show the number of unigenes, and one unigene may be associated with different GO terms. [file 12864_2020_6521_MOESM6_ESM.jpg]

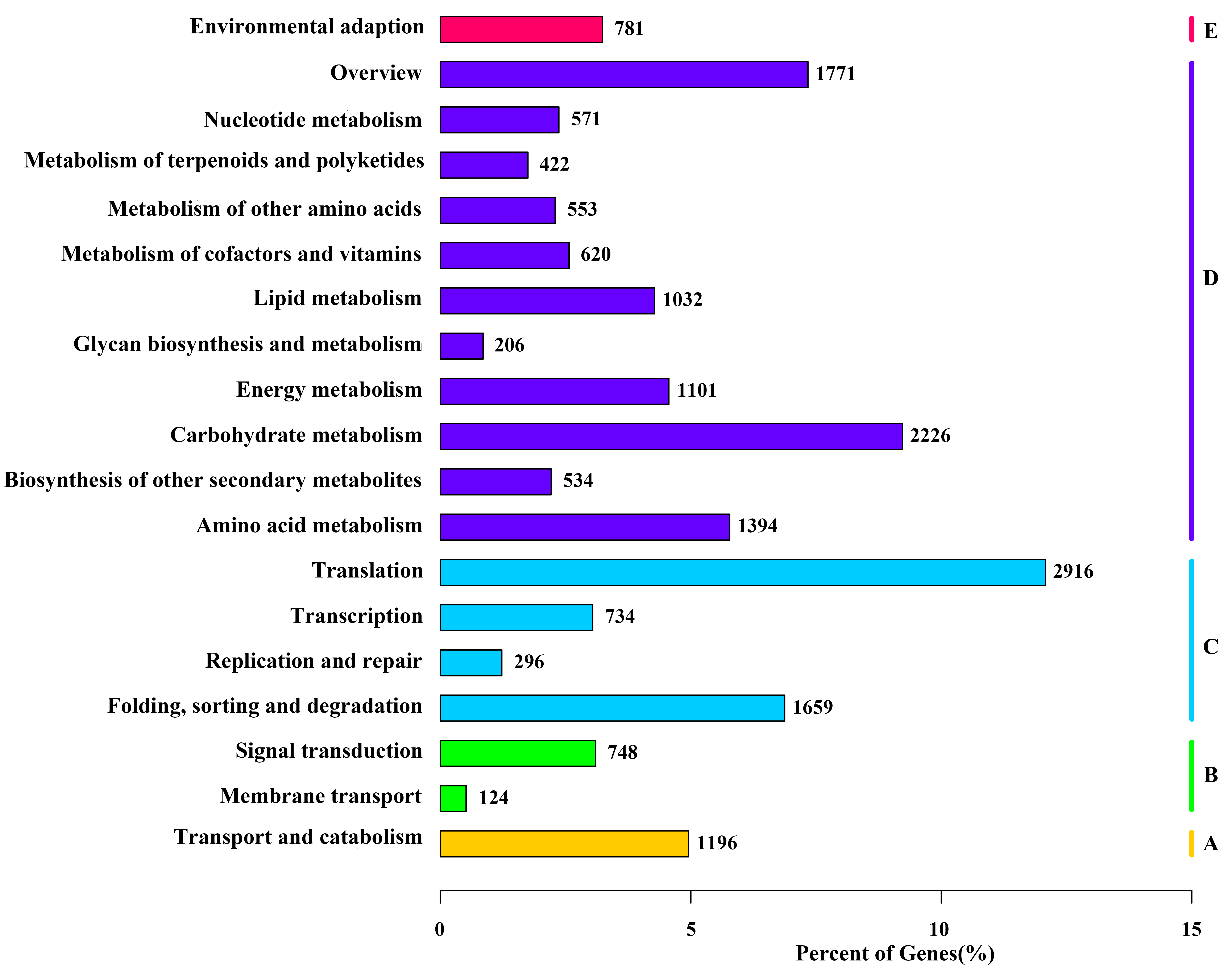

Supplement: Supplementary file 7 — Additional file 7 Figure S4. The KEGG pathway for the transcriptome of five major samples in Z. armatum. The unigenes were divided into five clusters according to KEGG metabolism pathways, A: Cellular processes, B: Environmental information processing, C: Genetic information processing, D: Metabolism, E: Organismal systems. [file 12864_2020_6521_MOESM7_ESM.jpg]

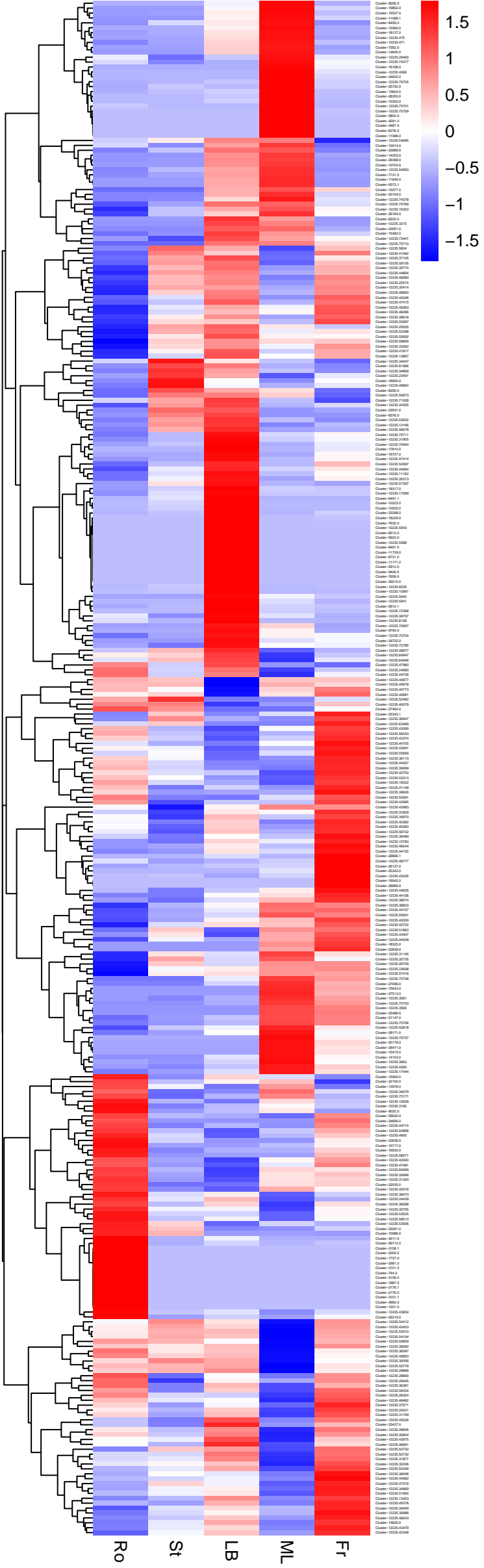

Supplement: Supplementary file 9 — Additional file 9: Figure S5. Heat map representation and hierarchical clustering of putative genes involved in fatty acid metabolism pathways. [file 12864_2020_6521_MOESM9_ESM.pdf]

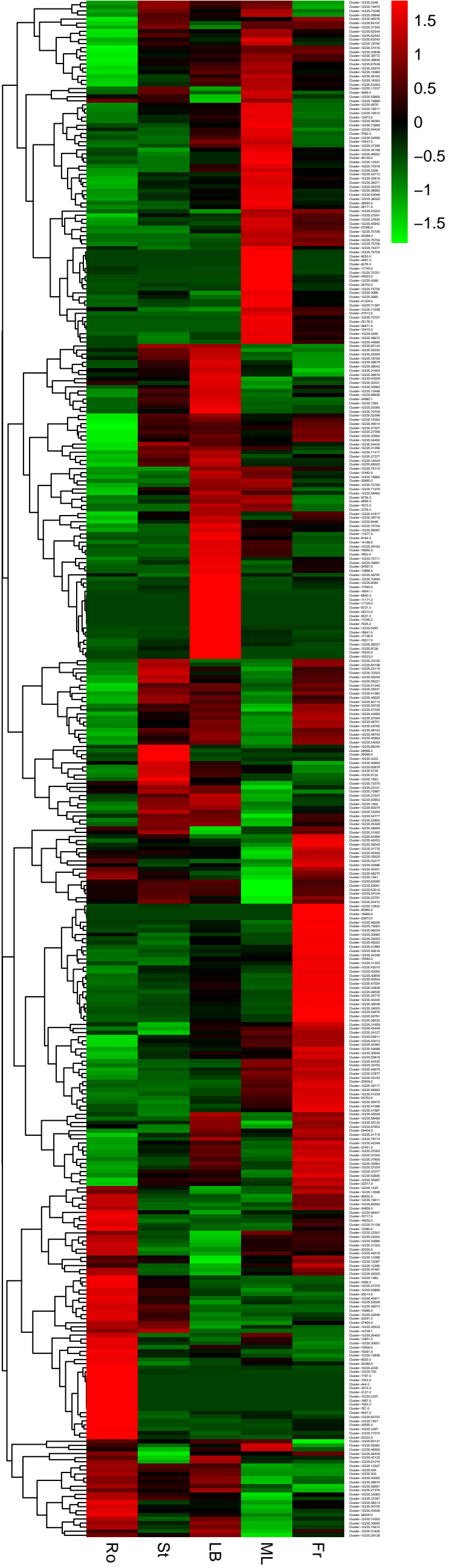

Ro St LB ML Fr

Supplement: Supplementary file 10 — Additional file 10: Figure S6. Heat map representation and hierarchical clustering of putative genes involved in terpenoid compounds biosynthesis pathways. [file 12864_2020_6521_MOESM10_ESM.pdf]

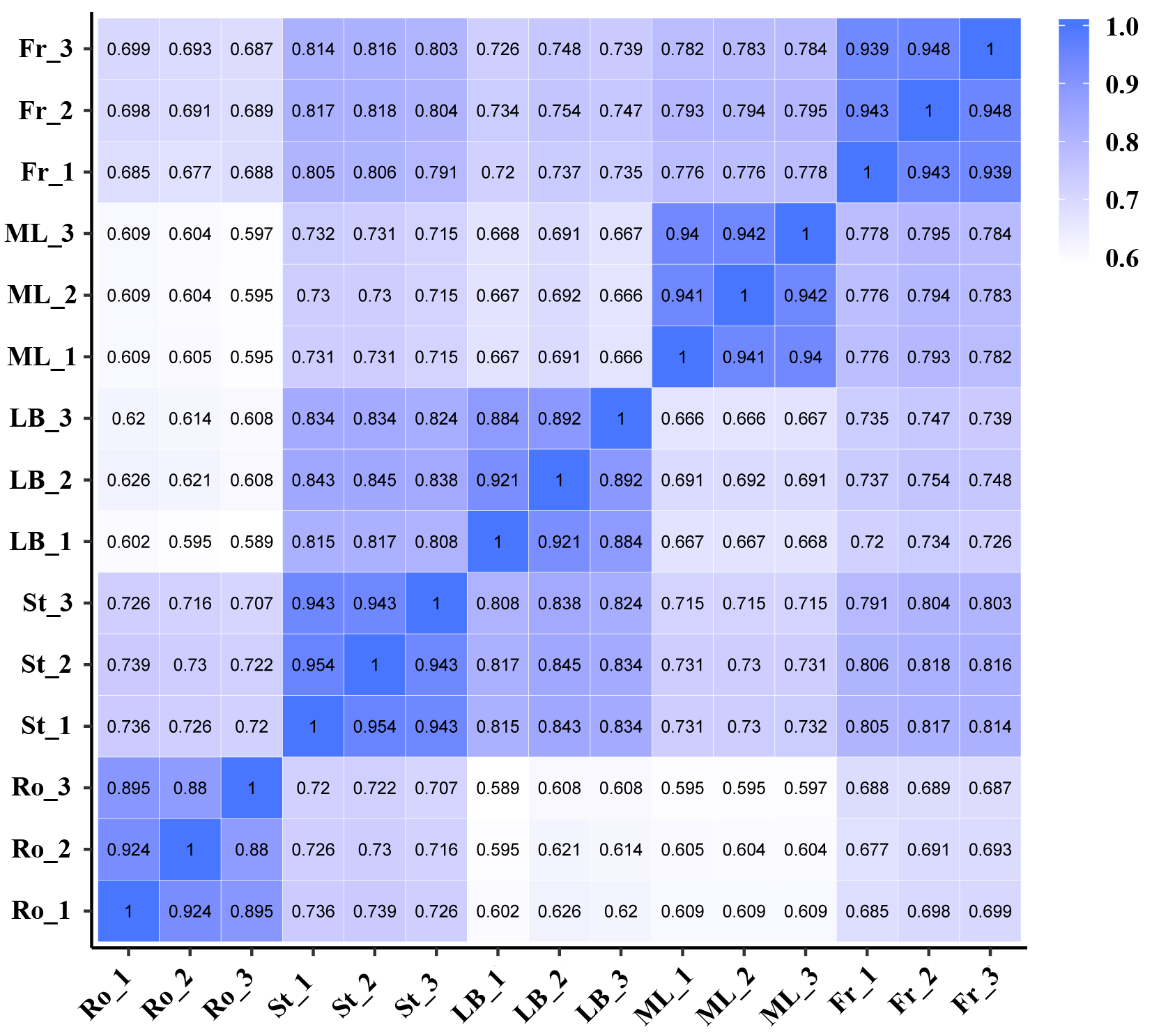

Supplement: Supplementary file 11 — Additional file 11: Figure S7. The pearson correlation coefficient (R2) was used to estimate the difference between the replicates of each tissue. The number between these two samples is given in the plot. The color represents R value, which shows high correlation in blue between two samples, while low correlation in white. [file 12864_2020_6521_MOESM11_ESM.png]

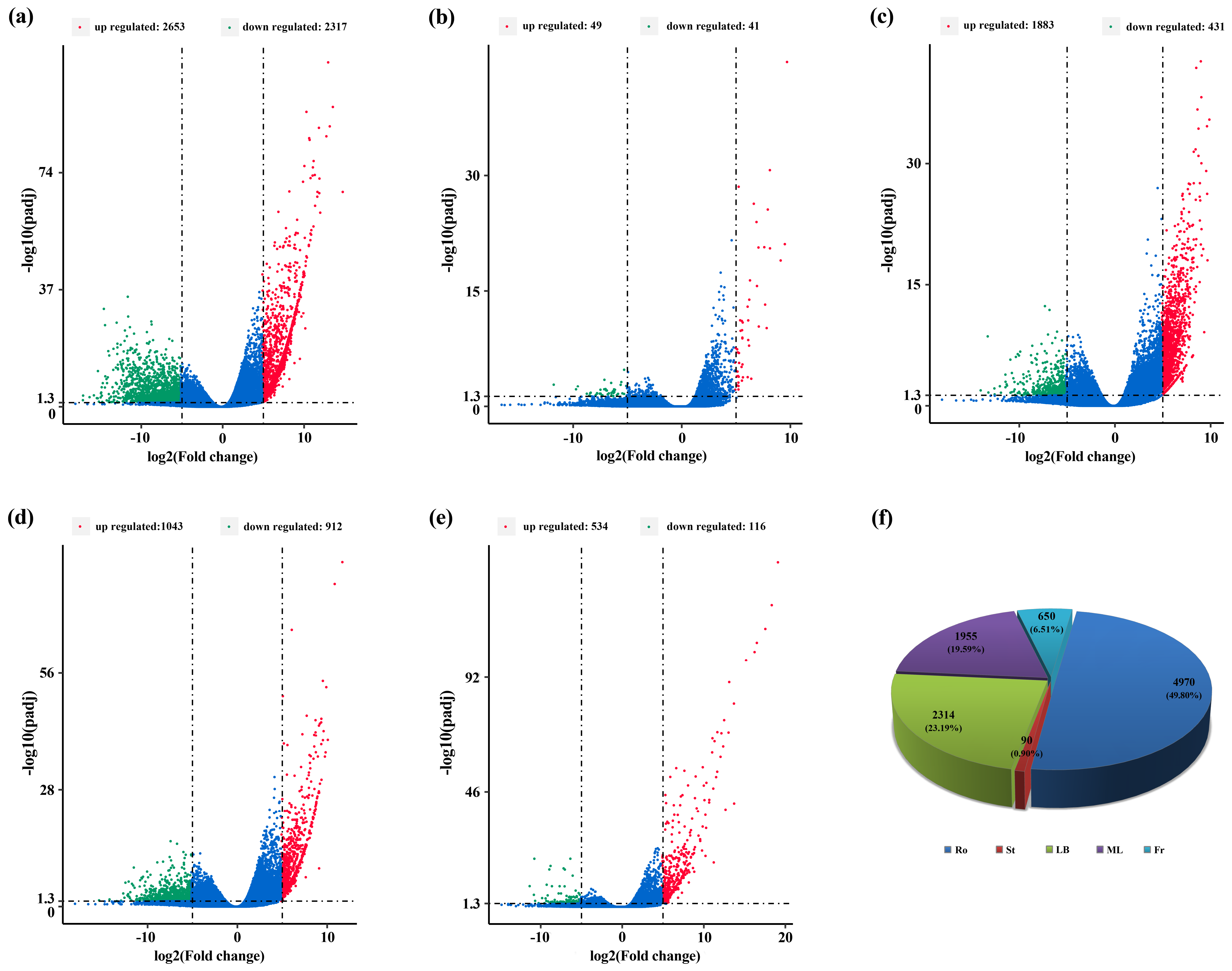

Supplement: Supplementary file 12 — Additional file 12: Figure S8. The volcano plot of differential expressed genes (DEGs) associated with organ-specific unigenes. (a)-(e) represent the organ-specific unigenes in Ro, St, LB, ML and Fr, respectively. The abscissa shown the fold change of DEGs in each comparison and the ordinate shows the significance of DEGs, q < 0.05. The red dot was up regulation, the blue dot was down regulation, and the green dot was not significant difference. (f) was the percentage of unigenes expressed in each organ. [file 12864_2020_6521_MOESM12_ESM.png]

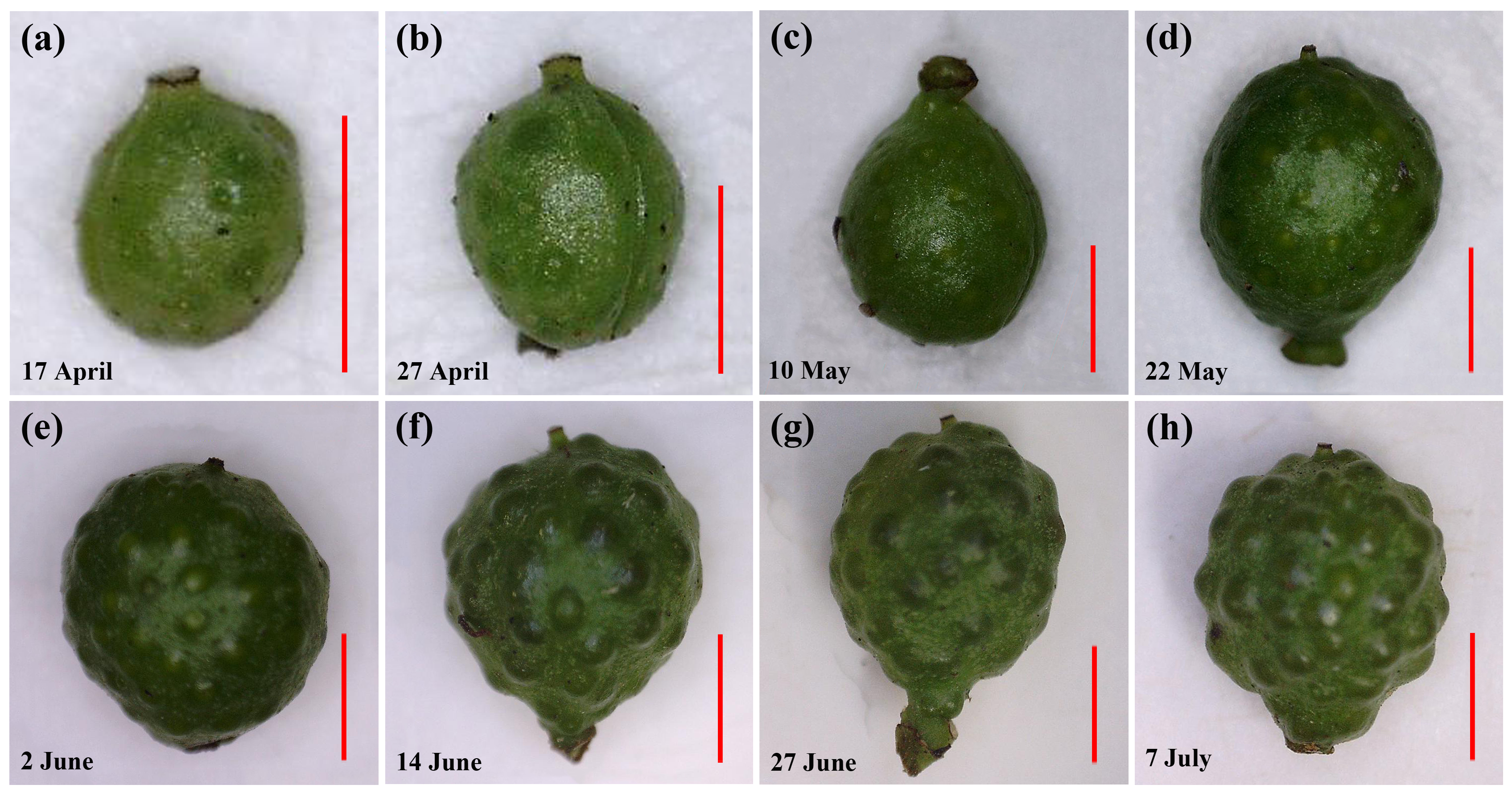

Supplement: Supplementary file 17 — Additional file 17: Figure S10. The samples involved in fruit development and maturation in Z. armatum. (a)-(h) represent the samples collected from Fr1 to Fr8, respectively. All the fruit samples were harvested from April to July 2019. The bar is 2 mm. [file 12864_2020_6521_MOESM17_ESM.png]

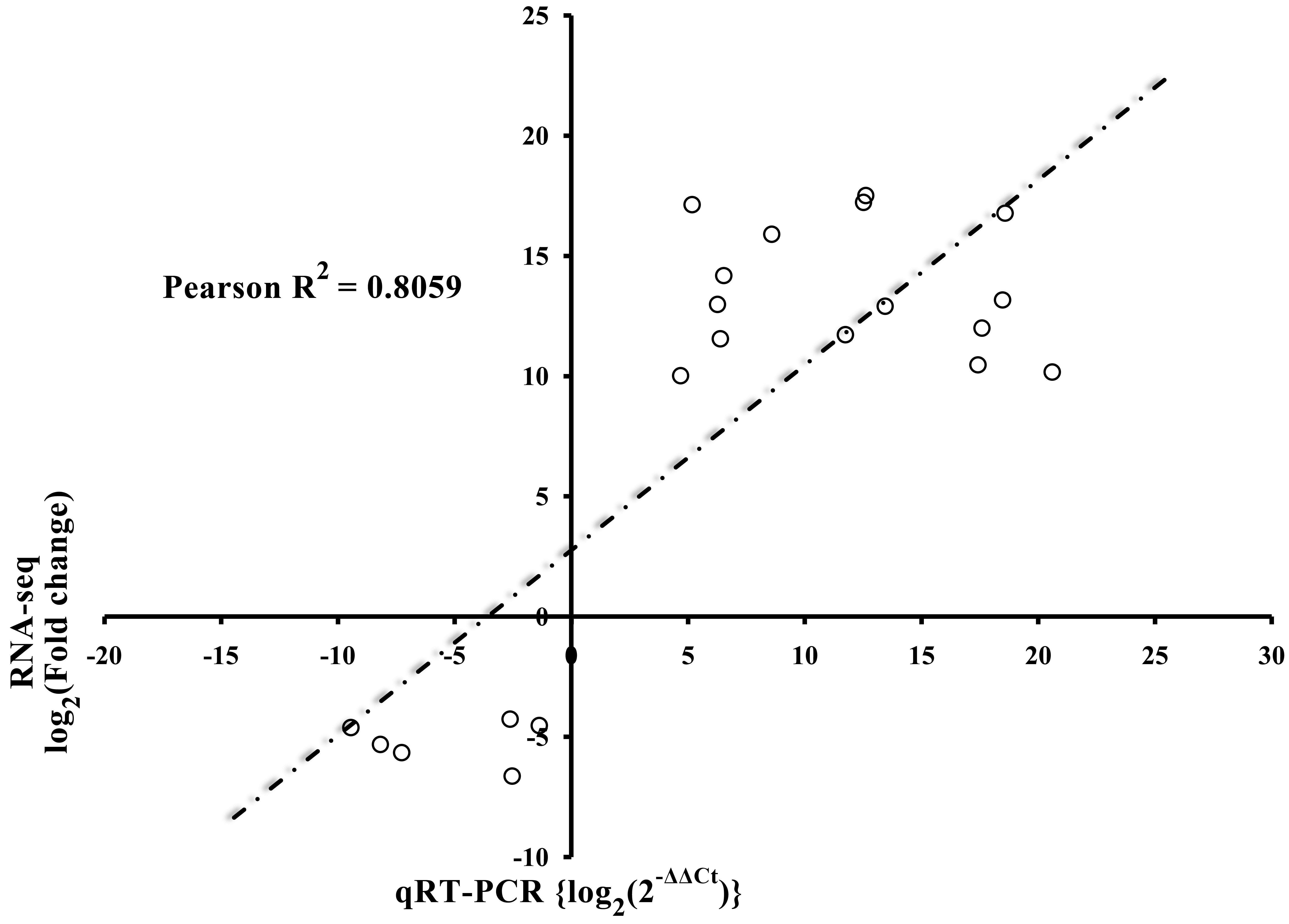

Supplement: Supplementary file 19 — Additional file 19: Figure S11. The correlation between qRT-PCR and RNA-seq data. Correlation between qRT-PCR and RNA-seq data of eight DEGs (21 terms) selected in Frvs. Ro, Frvs. St, Frvs.LB, Frvs.ML, LBvs. Ro, MLvs.LB, MLvs. Ro, and MLvs. St, including 6 down-regulation and 15 up-regulation. Pearson correlation coefficient = 0.8059 (p < 0.05). [file 12864_2020_6521_MOESM19_ESM.png]
